# Revealing the essential role of the lid in mclPHA intracellular depolymerase from *Pseudomonas putida* KT2440

**DOI:** 10.1007/s00253-025-13605-z

**Published:** 2025-10-07

**Authors:** Laura Isabel de Eugenio, José Daniel Jiménez, Elena Ramos, Lara Serrano-Aguirre, Jesus M. Sanz, M. Auxiliadora Prieto

**Affiliations:** 1 Polymer Biotechnology Group, Centro de Investigaciones Biológicas Margarita Salas (CIB-MS), CSIC. C/Ramiro de Maeztu, 9, 28040 Madrid, Spain; 2 Protein Engineering Against Antimicrobial Resistance Group, Centro de Investigaciones Biológicas Margarita Salas (CIB-MS), CSIC. C/Ramiro de Maeztu, 9, 28040 Madrid, Spain; 3 Interdisciplinary Platform of Sustainable Plastics towards a Circular Economy- Spanish National Research Council (SusPlast, CSIC), Madrid, Spain

**Keywords:** PHA depolymerase, Random mutagenesis, Enzyme engineering, Bioplastic recycling

## Abstract

**Abstract:**

Polyhydroxyalkanoates (PHAs) are microbial polyesters that serve as intracellular carbon reserves and represent promising biodegradable alternatives to conventional plastics. However, their large-scale application requires not only cost-effective production but also efficient strategies for recovery and recycling. Unlike short-chain-length PHAs, which are widely degraded by diverse enzymes, the intracellular degradation of medium-chain-length PHAs (mclPHAs) appears to be a genus-specific trait of *Pseudomonas*. In this context, the PhaZKT depolymerase from *Pseudomonas putida* KT2440 is considered a model enzyme for intracellular mclPHA mobilization; it is highly substrate-specific, acting almost exclusively on mclPHAs, and consists of an α/β-hydrolase fold with a lid domain, similar to lipases and other enzymes acting on lipid substrates, in contrast to extracellular PHA depolymerases, which generally lack this lid structure. Here, we explored the essential role of this lid structure through site-directed deletions and random mutagenesis. Targeted deletions within or near the lid completely abolished enzyme activity, highlighting its critical structural and functional importance. Random mutagenesis identified two beneficial variants: S184F, located in the lid hinge region, and G286R, situated in a still unmapped region. The S184F mutant exhibited increased esterase activity on *p*-nitrophenyl esters but significantly reduced depolymerase activity on mclPHA nanoparticles, indicating that lid integrity and dynamics precisely control substrate specificity and access. Molecular dynamics simulations supported these findings, revealing enhanced rigidity near the lid region in the S184F variant. Conversely, G286R showed substantially improved depolymerase activity toward mclPHA, suggesting alternative regions for beneficial mutations without compromising lid functionality. These results underscore the delicate balance between lid integrity and enzyme performance, offering insights into targeted protein engineering for optimized enzymatic recycling of bioplastics.

**Key points:**

• *The lid in PhaZKT is essential for depolymerase activity*

• *All lid-targeted mutants completely lost enzymatic activity*

• *Random mutagenesis identified two active distal mutants*

**Supplementary Information:**

The online version contains supplementary material available at 10.1007/s00253-025-13605-z.

## Introduction

Polyhydroxyalkanoates (PHAs) are a diverse family of microbial polyesters synthesized by numerous bacterial species as intracellular carbon and energy storage compounds. Their unique combination of biodegradability, biocompatibility, and stereoregularity (particularly their isotactic, chiral configuration) renders them highly attractive for a wide range of applications, from packaging to biomedical materials (Gregory et al. 2022). Among bioplastics, PHAs are unique in that they are both produced and degraded entirely by microbial systems, offering a naturally circular lifecycle that aligns with the principles of a sustainable bioeconomy (Rosenboom et al. 2022; Jiménez et al. 2024). Their broad structural diversity, which includes short-, medium-, and long-chain-length monomers, enables precise tuning of mechanical and thermal properties to suit specific end uses. However, despite their inherent biodegradability, not all PHA variants degrade uniformly under the same environmental conditions. Differences in monomer composition, crystallinity, and the incorporation of additives such as plasticizers can significantly influence their susceptibility to microbial degradation. This variability can compromise their environmental performance if uncontrolled release occurs, particularly in mixed formulations or complex waste streams (Narancic et al. 2018). As the global PHA market is projected to exceed 50,000 metric tons by 2025 (Rekhi et al. 2022), it is increasingly urgent to shift from a linear use model to a circular management strategy (Serrano-Aguirre and Prieto 2024). Among the available recycling options, enzymatic depolymerization offers notable advantages. Mechanical recycling, while operationally simple, suffers from limited recyclability due to the progressive loss of material properties with each cycle. Chemical recycling, on the other hand, typically relies on organic solvents and elevated temperatures, raising concerns regarding environmental sustainability (Vu et al. 2020). Enzymatic recycling represents a greener alternative, as it can be carried out under relatively mild conditions, often in aqueous systems, minimizing energy input and the generation of hazardous by-products (Pardo et al. 2025). However, the success of enzymatic strategies depends on the availability of robust and efficient enzymes capable of acting not only on the polymer backbone but also on additives such as esters commonly found in plastic formulations. In this context, protein engineering emerges as a critical approach for tailoring enzymatic performance to industrial demands, and it has become an essential tool in designing industrially viable end-of-life solutions for bioplastics. This has been exemplified in recent advances in polyethylene terephthalate (PET) recycling, where engineered PET hydrolases (PETases) have dramatically improved degradation efficiency and substrate scope (Liu et al. 2023).

**Fig. 1:**
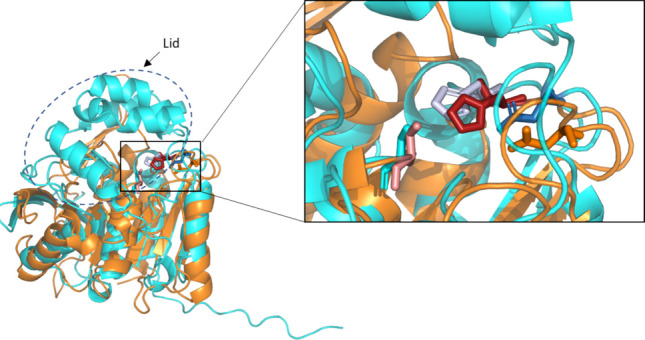
Structural alignment of two mcl-PHA depolymerases: intracellular PhaZ from *P. putida* KT2440 (cyan cartoon) and extracellular enzyme from *Pseudomonas solani* GK13 (orange cartoon). Catalytic triads are emphasized using stick representation. In PhaZKT, the catalytic residues (Ser102, Asp221, and His248) are depicted in cool tones, while in PhaZGK13 (Ser172, Asp228, and His260), they are represented in warm tones. Both proteins are displayed as semi-transparent cartoons (cartoon transparency 0.3) on a white background. Alignment was performed based on conserved core residues, with increased stick thickness to enhance clarity. The lid region is highlighted with a dashed oval

PHA depolymerases are specialized enzymes that catalyze the degradation of polyhydroxyalkanoates, playing a key role in the biological turnover of these polymers (Jendrossek and Handrick 2002). They are classified into extracellular and intracellular types based on their cellular localization and exhibit substrate specificities toward either short-chain-length (scl) or medium-chain-length (mcl) PHAs. While enzymes degrading scl-PHAs are widespread across many bacterial taxa, those capable of degrading mcl-PHAs are less common and have been predominantly identified in *Pseudomonas* species, although a few extracellular mcl-PHA depolymerases have also been reported in other genera, such as *Streptomyces* (García-Hidalgo et al. 2012; Martínez et al. 2015). Specifically, intracellular depolymerization of mcl-PHAs has so far been attributed exclusively to *Pseudomonas* species. This genus-specific specialization underscores the importance of characterizing *Pseudomonas* mcl-PHA depolymerases, which serve as model systems for studying intracellular PHA mobilization. Within this framework, the intracellular mcl-PHA depolymerase from *Pseudomonas putida* KT2440 (PhaZKT) has become a reference model for studying PHA degradation (de Eugenio et al. 2007, 2010). However, despite its biological relevance, the wild-type enzyme presents limitations that hinder its industrial application, such as low thermostability and modest esterase activity. In contrast, extracellular mcl-PHA depolymerases generally show higher stability and broader substrate specificity, and interestingly, they lack the lid structure that covers the active site in PhaZKT (Fig. 1). This suggests that the lid could be a key structural determinant of enzyme activity. In this study, the role of the lid domain in PhaZKT was investigated through targeted mutations, followed by random mutagenesis and activity-based screening to identify improved variants and gain further insights into the structural determinants of enzyme function. Improving the catalytic performance of PhaZKT could enable its use in enzymatic recycling strategies aimed at promoting the circularity of bioplastics.

## Materials and methods

### Materials

Poly(3-hydroxyhexanoate-co-3-hydroxyoctanoate) (mclPHA, ∼ 5% C6 and 95% C8) was purchased from Bioplastech Ltd. (Ireland). Poly(butylene succinate) (PBS), poly(butylene adipate-co-terephthalate) (PBAT), and polylactic acid (PLA) were purchased from Kompuestos (Spain). Poly(3-hydroxybutyrate) (PHB) was supplied by Sigma-Aldrich. All other products were of analytical quality or high-performance liquid chromatography grade.

### Bacterial strains and media

*Escherichia coli* DH10B and M15 strains were used for plasmid propagation and protein expression, respectively (the latter was employed for constructs derived from the pQE32 vector). Unless otherwise stated, *E. coli* cells were grown in LB (Sambrook and Russell 2001) medium at 37 °C. Selection antibiotics, gentamicin (10 µg/ml), kanamycin (50 µg/ml), or ampicillin (100 µg/ml) were added when needed. Production of radiolabeled mclPHA was performed using *Pseudomonas putida* GPo1 (formerly *P. oleovorans* GPo1) as described in de Eugenio et al. (2007).

###  DNA manipulations and plasmid constructions

Plasmids and oligonucleotides used in this study are listed in Table 1. All DNA manipulations and molecular biology procedures were performed following standard protocols, as previously described (Sambrook and Russell 2001). DNA fragments were purified using the Gene Clean kit (Bio 101, Inc., Vista, CA). *E. coli* cells were transformed using either the RbCl method or by electroporation with a GenePulser system (Bio-Rad) (Dower et al. 1988).

**Table 1:** Plasmids and primers. List of plasmids and oligonucleotide primers employed for gene cloning and expression, including their relevant features and references

	Relevant features or sequence	Reference
**Plasmids**		
pIZ1016	Gm^R^, broad-host range expression, lacI^q^ and *P*_tac_	(Martínez-Pérez et al. 2004)
pQE32	Ap^R^, T5 promoter, lac operator	Qiagen
pREP4	Km^R^, LacI	Qiagen
pUC18	Ap^R^, cloning plasmid	(Sambrook and Russell 2001)
pPAZ3	pQE32 derivative containing *phaZ* gene from *P. putida* KT2442	(de Eugenio et al. 2007)
pUC32	pUC18 derivative containing *phaZS184F* gene	This study
pUC88	pUC18 derivative containing *phaZG286R* gene	This study
pQEZ32	pQE32 derivative containing *phaZS184F* gene	This study
pQEZ88	pQE32 derivative containing *phaZG286R* gene	This study
**Primers**		
PHAZ1	AAGAATTCTCTAGAGGGTATTAATAATGCCGCAACCCTATATTTTCAG	
TAPA3′	CATTACCGCACCGGCAGC	
TAPA5′	TGGCAGCTGTTCGCAGGGC	
ZKTfus3′	CGCAGAGCTCTCACCCCCCCGAGGC	
FNGIG5′	ACCCCCTTGCTGATCGGCGCCAACCTCGAG	
PHAZ2	CAGATATCAAGCTTGGCCGCAGCTGTTTCA	
FNGIG3′	CTCGAGGTTCGGGCCGATCAGCAAGGGGGT	
PHAZQE	CGGGATCCCGCAACCCTATATTTTCAGGAC	
YYWQLF5′	CGGCAAGCTGGGCGCAGGGCTCGGCTGG	
YYWQLF3′	CCAGCCGAGCCCTGCGCCCAGCTTGCCG	

For construction of the lid-deletion mutant, two PCR fragments were generated using *Pfu* polymerase and *P. putida* KT2442 genomic DNA as a template, which yields blunt-ended products. Fragment 1 was amplified with primers PHAZ1 and TAPA3, and fragment 2 with primers TAPA5′ and ZKTfus3. Prior to ligation, fragment 2 was phosphorylated using T4 polynucleotide kinase (New England Biolabs) in the presence of 1 mM ATP. After ligation, the resulting DNA sequence was inserted into the pIZ1016 plasmid, previously digested with *Sac*I and *Hin*dIII.

Plasmids bearing phaZKT with the deletion of specific small regions in the lid were constructed by overlap extension PCR using *P. putida* KT2442 genomic DNA as a template, generating two partially overlapping fragments flanking the region to be removed in each case. For the FNGIG loop, a deletion mutant was obtained by deleting residues 34–38 using primers FNGIG5′ and PHAZ2 (fragment 1) and FNGIG3′ and PHAZQE (fragment 2), and cloning the resulting product into pQE32. For the YYWQLF mutant, targeting residues 190–195, overlap PCR was performed using primers YYWQLF5′ and PHAZ2 (fragment 1) and YYWQLF3′ and PHAZQE (fragment 2), and the final construct was cloned into pQE32. The fragments were fused by PCR without external primers, followed by amplification of the full-length product using external primers (PHAZQE and PHAZ2), restriction digestion, and cloning into pQE32.

Standard PCR reactions were carried out using 2 units of AmpliTaq DNA polymerase (PerkinElmer Life Sciences) in the presence of 100 ng of template DNA, 1 µg of each dNTP, and 2.5 mM MgCl₂, following the manufacturer’s recommended buffer conditions. Annealing temperatures were adjusted based on the GC content of the primers used.

Plasmid constructs were confirmed by Sanger sequencing using the same primers employed during amplification. Sequencing was performed with dye-labeled terminators and analyzed on an ABI Prism 3730 DNA analyzer (Applied Biosystems Inc.).

### Random mutagenesis of the phaZKT gene

To obtain clones producing PhaZKT depolymerase with increased esterase activity, a library of mutants was generated using the error-prone PCR technique (Bulter et al. 2003). The reaction was carried out using a Mastercycler (Eppendorf) and the AmpliTaq™ DNA polymerase enzyme along with Buffer II, which lacks MgCl₂ (Applied Biosystems).

The reaction mixture included genomic DNA from *P. putida* KT2442 as a template (15 ng), oligonucleotides PHAZ1 and PHAZ2 (both at 0.5 μM) (Table 1), Buffer II without MnCl₂ (1×), MnCl₂ (0.2 mM), MgCl₂ (0.75 mM), DMSO (5%), DNA polymerase (50 U/ml), and a skewed dNTP concentration: dATP and dGTP (both at 0.2 mM) and dCTP and dTTP (both between 0.4 and 0.6 mM).

The amplification was performed under the following conditions: one cycle of 5 min at 95 °C, 1 min at 60 °C, and 2 min at 72 °C, followed by 30 cycles of 1 min at 95 °C, 1 min at 60 °C, and 1.5 min at 72 °C. Fragments were purified and cloned into the pUC18 vector, and the resulting ligation was transformed into *E. coli* DH10B. Positive clones were selected using the esterase activity assay in plate (see below). Genes from the clones with the highest activity were used to generate N-terminal 6-His–tagged versions of the mutant proteins by PCR amplification with primers PHAZQE and PHAZ2, followed by restriction digestion and cloning into the pQE32 vector.

### Protein expression, purification, and detection

As a preliminary approach, the activity of the different protein variants was assessed using soluble fractions from crude cell extracts. For this, cells were grown in the presence of inducer (1 mM IPTG) at 30 °C, harvested by centrifugation, disrupted using a French press, and centrifuged at 27,000 × *g* to separate the soluble fraction from cell debris.

For His-tag protein purification, *E. coli* M15 (pREP4, pPAZ3) and the clones producing mutant variants of PhaZKT were first grown overnight in LB medium supplemented with ampicillin and kanamycin. The following day, cultures were diluted to an optical density at 600 nm (A₆₀₀) of 0.1. Once they reached mid-log phase (A₆₀₀ ≈ 0.5), protein expression was induced with 0.1 mM IPTG, and cells were incubated for an additional 4 h at 30 °C.

Induced cultures (500 ml) were harvested and resuspended in 40 ml of lysis buffer (50 mM sodium phosphate, pH 8.0, 5 mM Tris–HCl, 20 mM imidazole, 300 mM NaCl). Cells were disrupted by passing the suspension through a French press at 1000 psi (four passes). Lysates were centrifuged at 27,000 × *g*, and the supernatant was applied to a 2 ml Ni–NTA agarose column (Qiagen) pre-equilibrated with washing buffer (same as lysis buffer, but with 75 mM imidazole). After extensive washing, proteins were eluted with 500 mM imidazole in the same buffer. Eluted fractions were dialyzed against 50 mM Tris-HCl pH 8.0, 300 mM NaCl using PD-10 desalting columns (GE Healthcare). Desalting of the proteins to remove imidazole from the purification buffer is essential for measuring activity toward *p*NP derivatives, as imidazole can catalyze the hydrolysis of *p*-nitrophenyl esters by forming N-acylimidazole intermediates (Bender and Turnquest 1957). However, the removal of imidazole may affect enzyme activity toward other substrates or compromise its stability (Hamilton et al. 2003). All steps were conducted at 4 °C.

Sodium dodecyl sulfate polyacrylamide gel electrophoresis (SDS-PAGE) was performed using standard protocols (Sambrook and Russell 2001). For immunodetection, proteins were transferred onto PVDF membranes and visualized using the ECL™ Western Blotting Detection System (Amersham Biosciences), following the manufacturer’s guidelines. A polyclonal rabbit antiserum against PhaZ was previously generated using purified PhaZ protein as immunogen, as described in de Eugenio et al. (2007). For Western blot analysis, 2.25 μg of total protein from cell extracts was loaded onto SDS-PAGE gels and subsequently transferred to nitrocellulose membranes. Membranes were blocked overnight at 4 °C in PBS containing 5% (w/v) non-fat dry milk (10 mM sodium phosphate, pH 7.4, and 140 mM NaCl). After blocking, membranes were washed three times for 10 min in PBS supplemented with 0.1% Tween-20 (PBS-T) and then incubated for 1 h at room temperature with anti-PhaZ primary antibody diluted 1:10,000 in PBS-T. Following additional washes, membranes were incubated for 30 min at room temperature with HRP-conjugated anti-rabbit IgG (GE Healthcare) at a 1:10,000 dilution. Chemiluminescent signal was developed using the ECL reagents, and membranes were exposed to Hyperfilm MP (Amersham Pharmacia Biotech) for 1 min. Band intensity was quantified using Quantity One software (Bio-Rad).

Protein concentration was determined either by the Bradford assay (Bradford 1976) or, once purified, by measuring absorbance at 280 nm using an extinction coefficient of 46,410 M^−1^ cm^−1^.

### Enzyme activity assays

The enzymatic activity of wild-type PhaZKT and its variants was evaluated using multiple complementary assays designed to detect esterase, lipase, and depolymerase functions. Unless otherwise specified, standard conditions contained 50 mM Tris-HCl (pH 8.0) and 300 mM NaCl.

### Esterase and lipase activity assays

Esterase activity was quantified spectrophotometrically using *p*-nitrophenyl (*p*NP) esters of fatty acids with chain lengths ranging from C2 to C8. Substrates (purchased from Merck-Millipore or Sigma-Aldrich) were prepared as 8 mM stock solutions in isopropanol and added to a reaction mixture containing 50 mM Tris-HCl (pH 8.0) at 0.8 mM final concentration. Enzymatic hydrolysis was monitored by measuring the release of *p*NP at 405 nm. One unit of esterase activity was defined as the amount of enzyme required to release 1 μmol of *p*NP per min, using an extinction coefficient of 15,200 M⁻^1^·cm⁻^1^.

Additional qualitative detection of esterase or lipase activity was performed by halo formation on agar plates containing 1,2,3-tributyrin or triolein, with or without Rhodamine B staining, as previously described (de Eugenio et al. 2007). Clear zones or fluorescent halos around colonies were taken as indicators of substrate hydrolysis.

Esterase activity of growing cells was performed by the esterase activity assay in plate. Cells were plated and grown for 12 h in the presence of 1 mM IPTG. Colonies were overlaid with a layer containing 0.5% agarose in 50 mM Tris pH 8, 350 μg/ml naphthyl acetate (stock at 20 mg/ml in acetone), 350 μg/ml naphthyl octanoate (stock at 20 mg/ml in acetone), and 1.4 mg/ml Fast Blue RR SALT (stock at 80 mg/ml in acetone). Within seconds, colonies with higher esterase activity developed a black coloration.

### Depolymerase activity with bioplastics

The activity of depolymerases on various bioplastics was assessed using nanoparticles (NPs) of each polymer as a substrate. Two distinct nanoparticle preparation methods were used, depending on polymer solubility:

- Nanoprecipitation (for mclPHA): mclPHA was dissolved in acetone at 2 mg/mL. A total of 50 mL of this solution was added dropwise over 10 mL of cold water under stirring (350 rpm) using a separatory funnel. The resulting suspension was subjected to solvent evaporation at 60 °C using a rotary evaporator.
- Emulsion (for sclPHA, PLA, PBS): Polymers insoluble in acetone were dissolved in dichloromethane (DCM) at 20 mg/mL (3 mL). The solution was gently heated and stirred until fully dissolved. Then, 6 mL of an aqueous solution containing 0.1% (w/v) N-laurylsarcosine was added. The mixture was sonicated for 1 min (3 × 20-s pulses with 20-s rest intervals) to form a homogeneous emulsion. DCM was then evaporated at 60 °C with gentle agitation under a fume hood. In the case of sclPHA, polymer was dissolved at 6 mg/mL in 5 mL DCM and 15 mL of 0.01% (w/v) N-laurylsarcosine was added.

Final nanoparticle suspensions were adjusted to 10 mg/mL with water (except for sclPHA that was adjusted to 2 mg/mL) and stored at 4 °C. The average particle diameter ranged from 150 to 200 nm, with a polydispersity index of approximately 15%, as determined by dynamic light scattering (DLS) using a DynaPro MS/X instrument (Wyatt Inc.).

Enzyme reactions were carried out by incubating 200 μg of nanoparticle substrate with 1.8 μg of enzyme in 200 μL of standard buffer. The reaction was monitored by turbidity loss at 650 nm. One unit of enzymatic activity was defined as the amount of enzyme required to solubilize 1 μg of PHA per min, considering an apparent extinction coefficient for mclPHA of 1.44 µl·μg^−1^·cm^−1^ (de Eugenio et al. 2007). Additionally, PHA depolymerase activity plate assays were performed where 0.5 mg/ml NP was included in 1% agarose plates containing Tris–HCl 50 mM pH 8 and 300 mM NaCl.

For highly sensitive detection of depolymerase activity, a radiolabeled substrate assay was used, following the method described in de Eugenio et al. (2007). The assay employed 20 × concentrated soluble crude extracts obtained from IPTG-induced *E. coli* cultures expressing wild-type or mutant forms of PhaZKT. Reactions were monitored to detect the release of radiolabeled degradation products in the supernatants using a LKB Wallac liquid scintillation counter.

### Thermal stability assay

To assess thermal stability, enzyme samples were incubated at various temperatures for 10 min, followed by immediate cooling on ice for 5 min. Residual activity was then measured under standard assay conditions using *p*-nitrophenyloctanoate as the substrate.

### Structural modeling

The three-dimensional structures of PhaZKT and its mutant variants were predicted using AlphaFold, a deep learning-based algorithm for protein structure prediction (Jumper et al. 2021). The amino acid sequences were submitted to the AlphaFold 3.0 prediction server (https://alphafoldserver.com/), and the resulting models were evaluated for structural accuracy and reliability based on the confidence scores (pLDDT) and predicted aligned error (PAE). The generated structures were visualized and analyzed using PyMOL (Schrödinger, LLC 2015a; Schrödinger, LLC 2015b) to assess the integrity of the catalytic domain and potential conformational changes in the mutant variants.

### Molecular dynamics simulations

Homology models of the wild-type (PhaZKT) and mutant (S184F) proteins were used as starting structures. Simulations were performed using GROMACS 2021.5 with the CHARMM27 force field and TIP3P water model. Each structure was placed in a dodecahedral box with a 1.0 nm buffer from the edges and solvated with TIP3P water molecules. The systems were neutralized with Na⁺ and Cl⁻ ions.

Energy minimization was carried out using the steepest descents algorithm until maximum force was below 1000 kJ/mol/nm. Equilibration followed a two-step protocol: NVT (300 K, 8 ps) with position restraints on heavy atoms using the V-rescale thermostat (Bussi et al. 2007), and NPT (300 K, 1 bar, 500 ps) using the Parrinello–Rahman barostat (Parrinello and Rahman 1981).

Production runs were conducted for 10 ns without restraints, using periodic boundary conditions. Key parameters included a 2 fs integration step, Particle Mesh Ewald for long-range electrostatics (Darden et al. 1993), and the LINCS algorithm for bond constraints (Hess et al. 1997). Simulations were run on CPU/GPU hybrid nodes when available.

Trajectory quality was monitored through RMSD, temperature, pressure, and total energy stability. Final structures were extracted and visualized in PyMOL to confirm the absence of artifacts.

## Results

### Targeted lid mutations of PhaZKT compromise enzyme activity

To explore whether the absence of the lid domain could be related to the better properties shown by extracellular mclPHA depolymerases, such as more robustness and less substrate specificity, targeted mutations were introduced to modify the PhaZKT lid domain. These mutations aimed to either completely delete the lid structure or alter its conformation by modifying key segments. The effect of these variations on the activity of the intracellular depolymerase was assessed through a plate-based esterase assay where an insoluble brown product formed that precipitates at the reaction site, allowing the visual detection of the clones with augmented esterase activity.

**Fig. 2:**
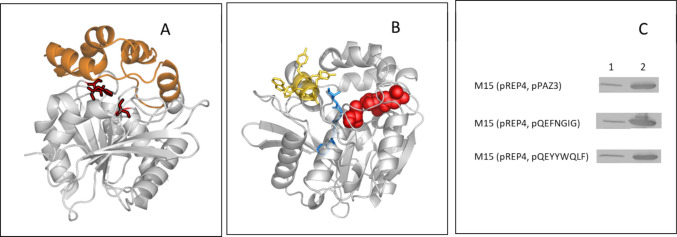
Structural representation of the PhaZ enzyme from KT2442 highlighting key functional regions. **A** Lid region of PhaZKT. The protein is shown as a semi-transparent cartoon in grey, with the catalytic triad (Ser102, Asp221, His248) represented in red sticks to indicate the configuration of the active site. The region encompassing residues 133–192 is highlighted in orange and corresponds to the lid domain that has been deleted in the truncated variant. **B** Specific regions encompassing lid functionality in PhaZKT. The protein is displayed as a semi-transparent cartoon in grey, with key regions emphasized in distinct colors. The catalytic triad (Ser102, Asp221, His248) is shown in red as sticks and spheres to indicate the spatial arrangement of the active site residues. Two regions subjected to mutagenesis are highlighted: residues 34–38 (FNGIG) in marine blue and residues 190–195 (YYWQLF) in gold. The blue loop (34–38) connects the lid domain with the catalytic core; its removal may disrupt the interactions between these domains, potentially leading to lid opening. The segment 190–195, a hydrophobic hinge region, may further modulate the structural stability of the enzyme, acting as a secondary connection between the lid and the main domain. **C** Comparative Western blot analysis of PhaZKT expression in *E. coli* after deletion of the FNGIG or YYWQLF loops. The production of the wild-type enzyme was compared to that of the mutant variants. Lanes: 1, soluble fraction; 2, insoluble fraction

A lid-deletion mutant of PhaZKT was generated through blunt-end ligation of PCR fragments flanking the lid region (Fig. 2A). Unfortunately, the plate-based esterase assay with this strain yielded negative results. The loss of activity may reflect partial unfolding or misalignment of key catalytic elements in the absence of the lid, but it can also indicate a general loss of function, as the mutant showed no activity even against its natural substrate, PHA. This was confirmed using a highly sensitive radiolabeled mclPHA assay, which showed no detectable depolymerase activity when assaying soluble cell extracts. Consequently, a more targeted approach was employed to destabilize the lid domain by mutating specific regions. The 34–38 loop (FNGIG) could connect the lid to the main domain, and its deletion would potentially disrupt interactions between these domains, leading to lid opening (Fig. 2B). Additionally, the deletion of segment 190–195 (YYWQLF), which could function as another hinge notably hydrophobic, would reduce structural stability and potentially expose the catalytic site. These mutations were introduced separately to evaluate their specific impact on enzyme structure and activity. Unfortunately, mutants targeting specific regions exhibited no detectable activity over *p*-nitrophenyl esters or mclPHA, indicating that the structural alterations were also too disruptive to maintain functional integrity. To verify that the lack of activity was not due to insufficient protein expression, we performed Western blot analysis on the soluble fractions. The expression levels of the mutant proteins were comparable to those of the wild-type enzyme, indicating that the mutations did not affect overall protein production (Fig. 2C).

In light of these observations, we redirected our efforts toward random mutagenesis and activity-based screening, aiming to identify subtle, structurally tolerated mutations capable of modulating substrate specificity without compromising catalytic function.

### Random mutagenesis reveals mutations enhancing esterase activity in PhaZKT

**Fig. 3:**
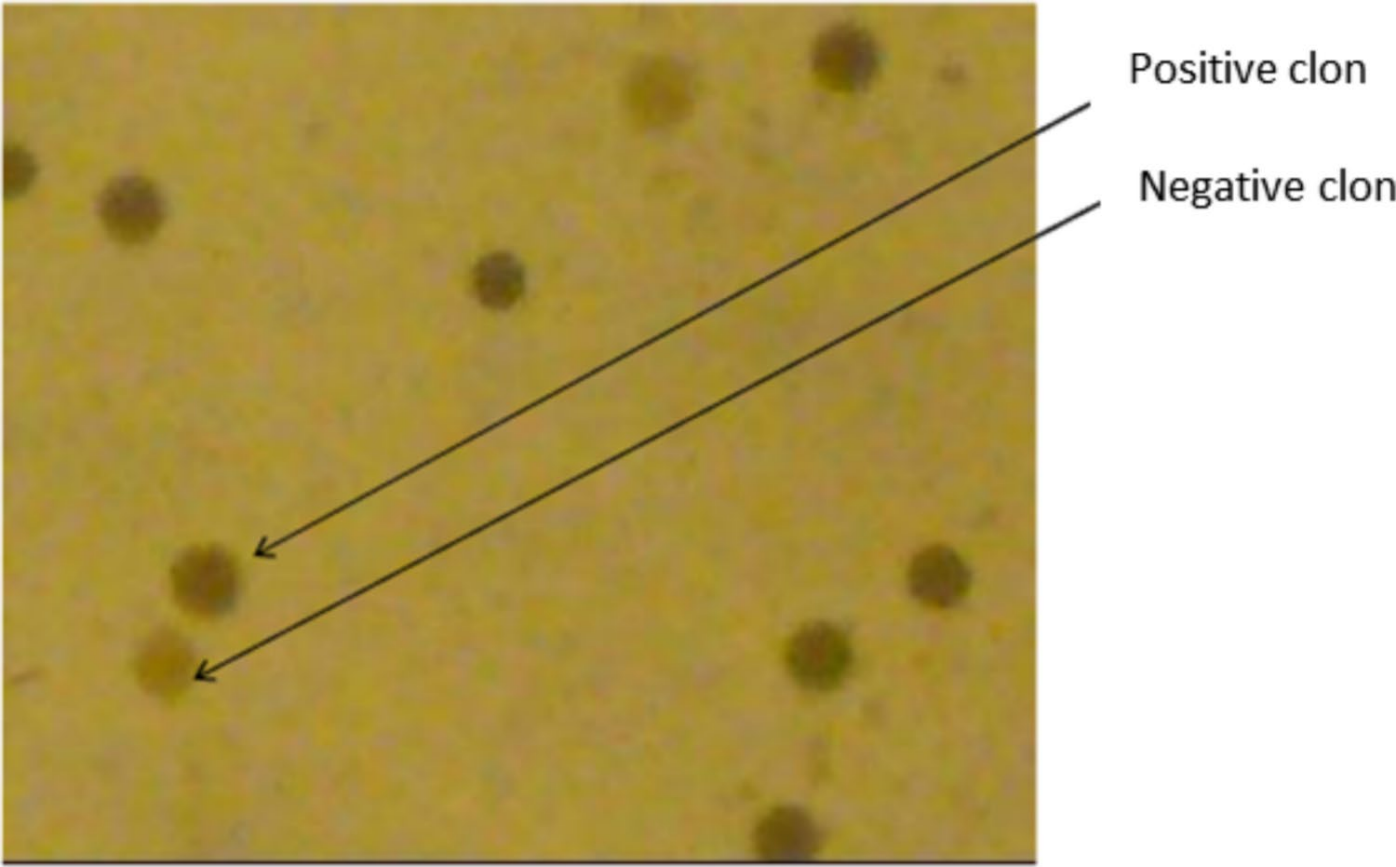
Esterase activity assay on agar plate. Clones from error prone PCR clones were grown on ampicillin LB plates and cover with an overcoat of naphtyl acetate and octanoate in Fast blue RR salt agarose. Positive clones quickly developed dark color

The best amplification results for error-prone PCR were obtained in the presence of 0.6 mM pyrimidine bases with DMSO or 0.4 mM pyrimidine bases without DMSO. From these mutant libraries, approximately 4,940 colonies were obtained. Screening with a plate-based esterase assay identified ~ 400 positive clones (~ 8%), which were re-plated on LB-ampicillin-IPTG plates. About 100 colonies grew under these conditions, all of which tested positive upon re-screening. With this method, two clones with the highest esterase activity were selected (Fig. 3), and their *phaZ* genes were sequenced (*phaZG286R* and *phaZS184F*).

**Fig. 4:**
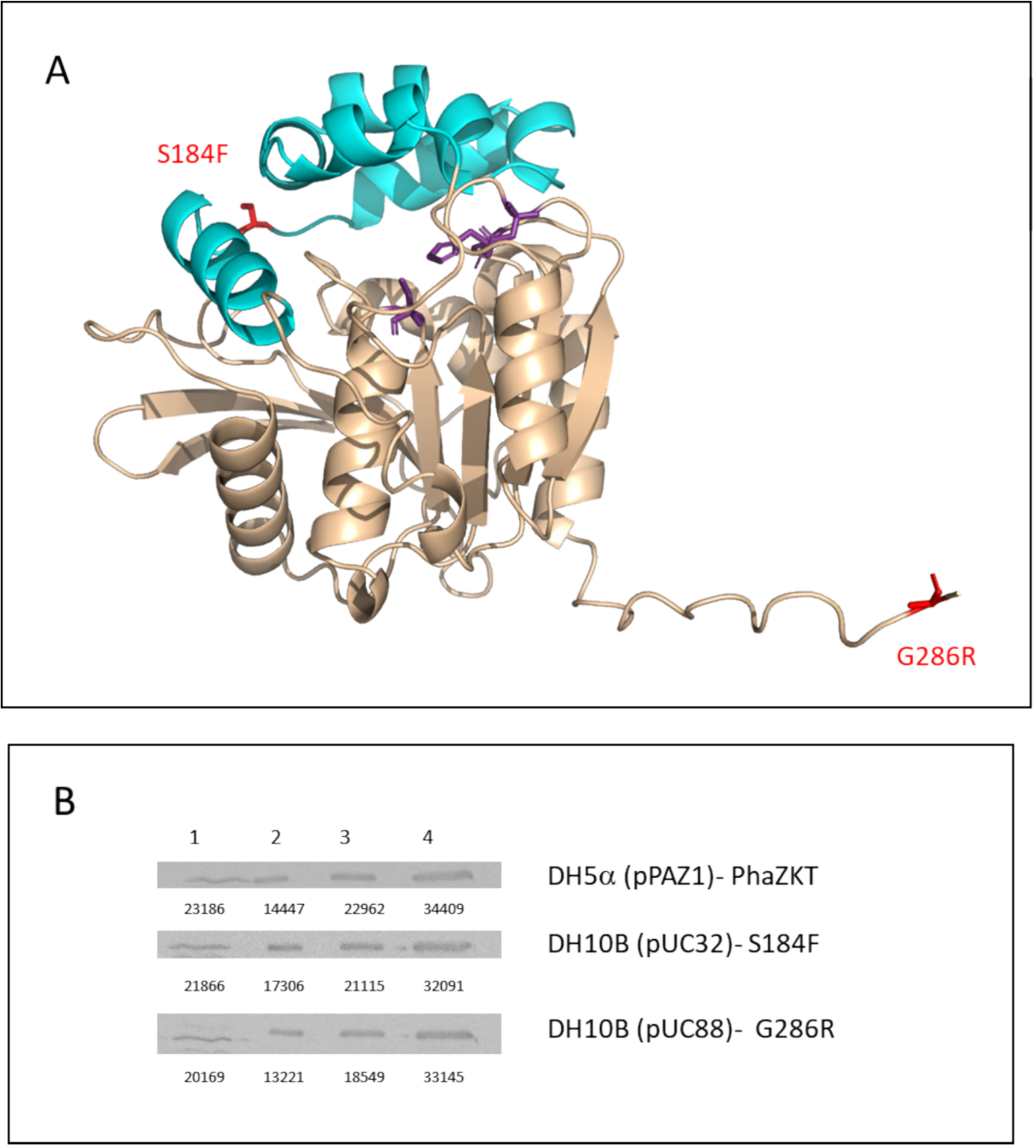
**A** Homology model of PhaZKT showing the position of the point mutations identified in variants S184F and G286R. G286R mutation is located in the C-terminal region, while S139F replacement lies at the interface between the lid domain (cyan) and the catalytic core (grey). Catalytic triad residues are shown in purple, and the mutated residues are highlighted in red. **B** Semiquantitative western blot analysis of PhaZ depolymerase production and its variants. Lane 1: supernatant of the soluble extract from the corresponding strain: a total of 320 ng of each supernatant was loaded; lane 2: 140 ng of purified PhaZKT; lane 3: 350 ng of purified PhaZKT; lane 4: 700 ng of purified PhaZKT Numbers shown below each band indicate the densitometric values obtained

In both sequences, a single nucleotide variation was observed (Fig. S1). The first clone showed a Gly286Arg change, while the second clone bears a Ser184Phe substitution (Fig. 4). Interestingly, the S184F mutation is located in the hinge region between the lid and the core of the protein. Although our rational attempts to modulate activity through targeted lid deletions were unsuccessful, this randomly identified variant exhibited increased esterase activity, with the mutation positioned in a structurally relevant region, the hinge. In contrast, the G286R mutation is situated in a region far from the lid domain (in sequence) that could not be modeled (Fig. 4). Consequently, the structural impact of this mutation is more difficult to predict. This finding underscores the ability of random mutagenesis to uncover changes beyond the reach of rational design.

The change in the substrate specificity of these strains was preliminary assayed with soluble cell fractions obtained after cell disruption, centrifugation, and total protein normalization. The activity of both mutants, along with the wild-type-expressing strain as a control, was tested against mclPHA nanoparticles by turbidimetry, as described in the “Materials and methods” section. Under these conditions, only G286R exhibited detectable, albeit low, depolymerase activity. Consistently, previous studies reported that the wild-type enzyme present in the soluble fraction did not exhibit detectable depolymerase activity against this substrate, likely due to the low levels of soluble protein produced (de Eugenio et al. 2007). To increase the sensitivity, radiolabeled mclPHA nanoparticles suspensions were used. Using ^14^C-PHA nanoparticles, the activity of G286R was 83 μg PHA hydrolyzed·mg prot^−1^·min^−1^. The activity of S184F was lower (3.53 μg PHA·mg prot^−1^·min^−1^) but higher than that of PhaZKT (0.05 μg PHA·mg prot^−1^·min^−1^). To prove that the concentration of PhaZ was similar in all supernatants tested and that the increase in activity was not due to a higher enzyme concentration, a semi-quantitative Western blot was performed using known concentrations of purified PhaZKT as a standard for calibration. As shown in Fig. 4B, all clones showed a similar level of PhaZ in the supernatant.

The activity of PhaZ variants on other substrates, including tributyrin, triolein, trilaurin, trioctalein, tridecanolein, *p*-nitrophenylpropionate, *p*-nitrophenylbutyrate, *p*-nitrophenylvalerate, and *p*-nitrophenyloctanoate, was also measured. The wild type showed no detectable activity toward any triglyceride (including tributyrin) or *p*-nitrophenylalkanoate. Only the mutant variants displayed residual activity on tributyrin (data not shown). This may be due to the fact that the mutants do indeed possess higher typical esterase capacity, since esterases have been reported to be active against tributyrin but not triolein, whereas lipases can degrade triolein and tributyrin. However, the activity levels obtained against PHA and other substrates were consistently low across all assays, primarily due to the low levels of soluble protein in the crude extracts, as the cells predominantly formed inclusion bodies. Thus, a protocol was developed to purify the protein in the presence of His tag.

### Functional characterization of soluble PhaZKT mutants with enhanced esterase activity

PhaZKT and its mutant variants were predominantly expressed as inclusion bodies in *E. coli*. To enable purification and improve recovery, constructs encoding His-tagged versions of each protein (G286R and S184F) were generated. His-tagged PhaZKT, G286R, and S184F proteins accumulated in the insoluble fractions, being nearly undetectable in the soluble fraction (Fig. S2). However, since the wild type enzyme had been successfully purified from the soluble extract of M15 (pREP4, pPAZ3) strain (de Eugenio et al. 2007), mutant variants’ purification was attempted also from these strains’ soluble cell fractions.

Once the proteins were purified (Fig. S2B), their substrate specificity was assessed. The specific activity of wild-type and mutant variants was evaluated against a panel of *p*-nitrophenyl (*p*NP) esters of increasing acyl chain length (C2–C8). PhaZKT exhibited the highest activity against *p*-nitrophenyl propionate (C3), while *p*-nitrophenyl valerate (C5) consistently yielded the lowest activity, followed by *p*-nitrophenyl octanoate (Table 2). This pattern suggests that longer substrates, such as valerate and octanoate, may experience steric hindrance that limits efficient catalysis. Among the tested variants, S184F exhibited the highest overall esterase activity across the full *p*NP series, outperforming both the wild-type and G286R, and maintaining substantial activity up to the C8 acyl chain. G286R showed similar activity to PhaZKT regarding its activity on *p*NP-derivatives, although it is more active toward *p*-nitrophenyl octanoate, a trend that may be related to its superior performance on mclPHA.

**Table 2:** Relative hydrolytic activity of PhaZKT and its mutants against different *p*-nitrophenyl esters

	Relative activity (%)		
Substrates	PhaZKT	G286R	S184F
*p*-nitrophenyl acetate	86.4 ± 1.4	65.5 ± 0.3	81.2 ± 0.9
*p*-nitrophenyl propionate	96.0 ± 2.2	88.0 ± 2.5	100.0 ± 0.9
*p*-nitrophenyl butyrate	65.2 ± 2.2	62.6 ± 2.5	77.7 ± 2.1
*p*-nitrophenyl valerate	42.0 ± 3.1	49.4 ± 3.2	60.7 ± 3.6
*p*-nitrophenyl octanoate	54.2 ± 3.8	83.6 ± 3.4	80.6 ± 5.5

**Fig. 5:**
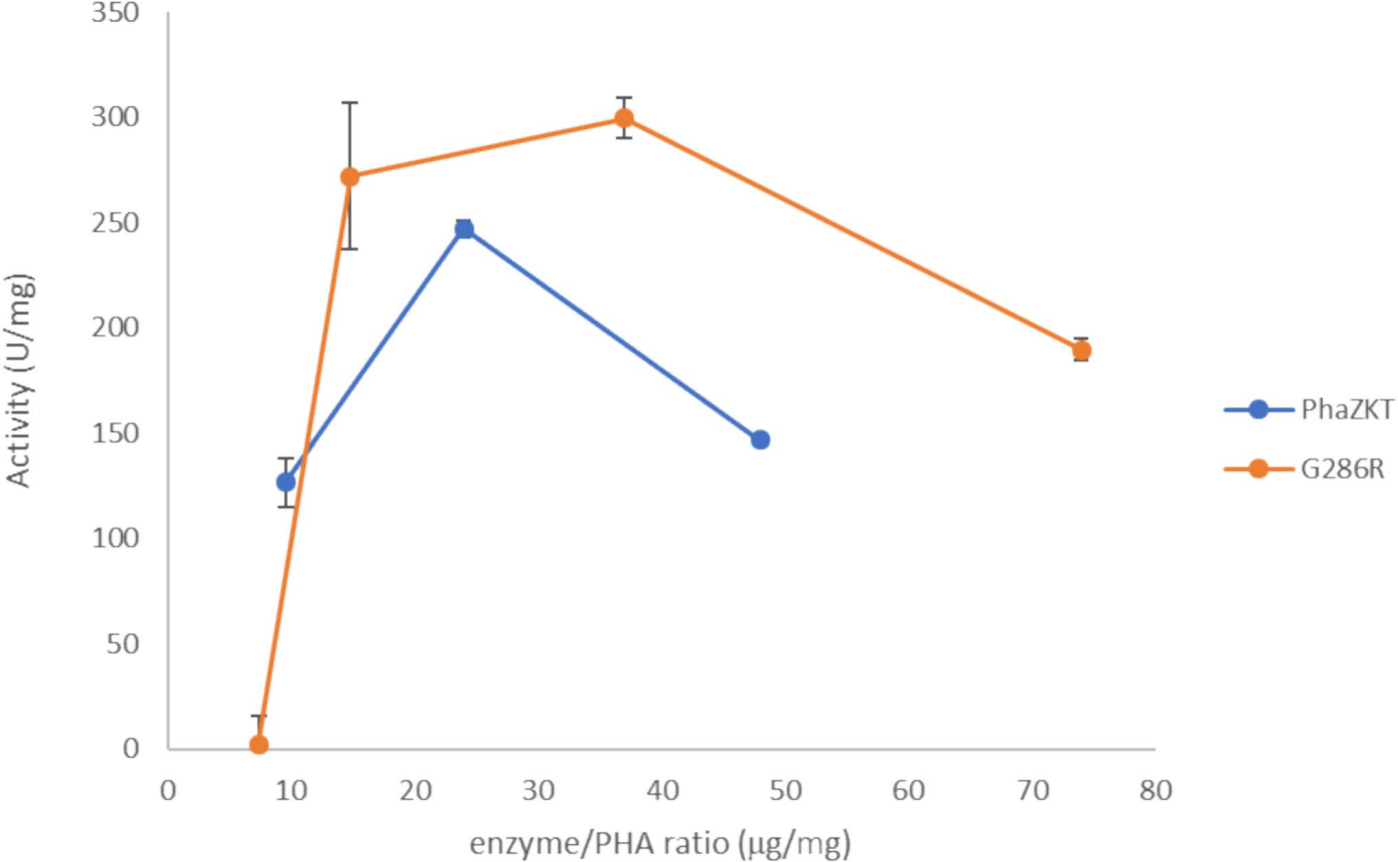
Depolymerase activity of PhaZKT and its G286R variant as a function of enzyme/polymer ratio. The G286R variant shows greater stability across the tested ratios, while PhaZKT exhibits a more pronounced variation in activity depending on the enzyme/polymer ratio. Each data point represents the mean of three independent experiments (*n* = 3), and the error bars indicate the corresponding standard deviation

Regarding activity on mclPHA, G286R showed the highest depolymerase activity (300 U/mg at a ratio enzyme/polymer of 37), 1.2-fold higher specific activity than PhaZKT (247 U/mg, at a ratio enzyme/polymer of 24), and 20-fold higher than S184F (15 U/mg, ratio enzyme/polymer 110), which exhibited a 16-fold reduction in activity compared to PhaZKT. Notably, the S184F variant exhibited almost no depolymerase activity while displaying increased esterase activity, highlighting how even a single mutation near the lid region can severely affect functionality, as seen with the targeted lid deletion mutants. In contrast to the S184F mutation, G286R displayed a slight increase in esterase activity alongside a marked enhancement in PHA depolymerase activity, despite the mutation being located far (in sequence) from the lid–active site region. The relative activity of the wild-type enzyme (PhaZKT) and the G286R variant as a function of the enzyme/PHB ratio is shown in Fig. 5. The wild-type enzyme shows a clear maximum at 24 µg PhaZ/mg PHA, followed by a decrease in activity at higher enzyme/PHB ratios, which may reflect inhibition due to excessive surface coverage. In contrast, the G286R variant reaches its maximum at around values between 14 and 40 µg/mg, given that the curve suggests the presence of a sustained maximum (plateau) across a broader range of enzyme/PHB ratios. Such behavior is consistent with the heterogeneous kinetic model proposed by Mukai et al. (1993) for PHB depolymerases, in which the activity profile reflects a balance between adsorption and catalytic turnover. This may indicate that the mutant is less sensitive to crowding effects on the polymer surface compared to the wild-type enzyme.

100% activity: 0.45 U/mg. Final concentration of *p*-nitrophenyl-fatty acids was 0.8 mM. Data represent mean ± standard deviation of triplicate experiments

To assess how detergents and cyclodextrins might affect these enzymes, we tested the impact of N-laurylsarcosine, a mild detergent, and methyl-β-cyclodextrin (MβCD), a compound known to modulate lipase activity by altering substrate accessibility or enzyme conformation (Ceynowa and and Koter 2001; Cao et al. 2017; Gutorov et al. 2024). Their influence was evaluated using mclPHA nanoparticles prepared by nanoprecipitation with no detergent. In the presence of 0.02% N-laurylsarcosine, the enzymes tested in this study retained 30% of their maximal activity (Table S1). When hydrolysis assays were conducted in the presence of 5% MβCD, the wild-type enzyme retained approximately 65% of its activity, the S184F mutant retained a similar level (~ 65%), while the G286R mutant retained only 40%. MβCD exerts a stronger inhibitory effect on G286R, and the observed differences between the two mutants may be related to the specific position of each mutation within the enzyme structure, making G286R more sensitive to the presence of MβCD. As the enzymes remained active despite partial inhibition by N-laurylsarcosine, their activity against other polymeric substrates whose nanoparticles cannot be prepared by nanoprecipitation was tested. PHB, PLA, PBS, and PBAT are insoluble in acetone and, thus, nanoparticles were prepared by emulsion using N-laurylsarcosine and dichloromethane. Under these conditions, neither the wild type nor the mutant variants exhibited detectable activity against these polymers. Finally, since high NaCl concentrations had previously been shown to be critical for PhaZKT activity, assays were conducted at both 300 mM (standard condition) and 60 mM NaCl to assess potential differences in the mutant variants. In all three enzymes, high salt concentration was equally essential for optimal activity, and no major differences were observed under these conditions (Table S1).

Preliminary thermal stability studies were performed by incubating the enzymes at temperatures ranging from 25 to 55 °C for 10 min, followed by activity assays using *p*-nitrophenyl octanoate (*p*NPO) as substrate. No apparent differences were observed between PhaZKT and G286R, both of which showed high sensitivity to temperature, retaining approximately 22% ± 2% and 24% ± 9% of their initial activity, respectively, at 37 °C, and only about 10% ± 3% and 13% ± 1% at 45 °C (Fig. S3). In contrast, S184F displayed improved thermal tolerance, about 51% ± 7% of its activity at 37 °C and 35% ± 19% at 45 °C, suggesting a slightly enhanced stability under elevated temperatures.

### Lid integrity influences PhaZKT activity: structural evidence from S184F

Understanding the structural basis for the altered function of the S184F variant is of particular interest, as this mutation leads to increased activity toward *p*-nitrophenyl esters while resulting in a loss of activity toward PHA substrates. Given its location, this mutation suggests that lid integrity is essential for PHA depolymerase activity. Located at the interface between the lid and core domains, the S184F substitution replaces a serine with a bulkier and more hydrophobic phenylalanine within the hinge region. This change could interfere with or destabilize local structural interactions, potentially affecting lid positioning or dynamics and leading to the observed activity pattern. To investigate this, molecular dynamics (MD) simulations were performed to compare the mutant with the wild-type PhaZKT.

Preliminary MD analyses of PhaZKT and S184F revealed substantial fluctuations in the C-terminal region of both models. This region (residues 266–283), which lacks defined secondary structure and is likely disordered, introduced noise that could hinder the interpretation of local structural changes near the mutation site. To minimize potential artifacts during the simulation, the poorly resolved C-terminal region (residues 266–283) was removed for MD simulations. This approach follows previous recommendations highlighting that intrinsically disordered terminal regions may introduce instability or noise in MD trajectories (Mani et al. 2023). Simulations were repeated using truncated versions of both proteins, excluding the terminal segment, in order to calculate and compare RMSD values. In these refined 10 ns trajectories, the RMSD profiles of the wild-type and mutant proteins were very similar, suggesting comparable global stability. However, RMSF analysis uncovered localized differences (Fig. S4): the mutated residue 184 displayed reduced fluctuation in S184F (ΔRMSF = + 19), indicating increased rigidity at the mutation site (Table S2). This local rigidification could reflect a conformational preference that facilitates access for small ester substrates, while potentially limiting the flexibility required for polymer degradation.

## Discussion

The interest in PHAs and their utility as substitutes for conventional plastics is beyond question, positioning them as key materials for a greener and more sustainable future (Rajvanshi et al. 2023). They are biodegradable, derived from renewable resources, and suitable for a wide range of applications. Beyond their role as sustainable plastics, PHAs also hold intrinsic value because all their incorporated monomers are chiral *R*-enantiomers, high-value compounds with potential uses in chemical synthesis and pharmaceutical manufacturing (Koller et al. 2010). This dual value, both as environmentally friendly materials and as sources of valuable chiral monomers, offers multiple avenues to offset production costs and enhance their industrial viability. The development of efficient methods to recover PHA waste and enzymatically depolymerize it into reusable monomers holds great promise for enhancing the economic viability of PHA-based materials. By lowering production costs and supporting a sustainable circular model, such depolymerization strategies could transform waste into a valuable resource and facilitate broader market integration. Consequently, there is strong interest in identifying or engineering improved depolymerases as catalysts for efficient PHA breakdown. However, while depolymerases acting on short-chain-length PHAs have been more extensively studied, those targeting medium-chain-length PHAs remain comparatively underexplored.

Intra-and extracellular mclPHA depolymerases display notable structural and functional differences. Two well-characterized model enzymes exemplifying these classes are those from *Pseudomonas putida* KT2440 (de Eugenio et al. 2007, 2008) and *Pseudomonas solani* GK13 (Schirmer et al. 1993; Schirmer and Jendrossek 1994), respectively. These enzymes share a common substrate, showing activity on mclPHAs both as native granules and in artificial polymer solutions, yet comparative analyses have highlighted mechanistic divergences between them (Jendrossek and Handrick 2002). Intracellular mclPHA depolymerase from *P. putida* KT2440 has been found to be less efficient in hydrolyzing soluble substrates such as *p*-nitrophenyl esters, displaying a more specific and restricted substrate profile, while the extracellular enzyme, on the contrary, is capable of hydrolyzing medium- to long-chain *p*-nitrophenyl esters. In addition to these functional differences, extra- and intracellular mclPHA depolymerases also exhibit clear structural distinctions. Although both enzymes adopt a typical α/β hydrolase fold with a β-sheet core surrounded by α-helices, the intracellular variant possesses a lid-like structure capping the common hydrolase fold (Fig. 1), formed by three α-helices located between the β + 1 and β + 2 loops. This structural feature is characteristic of lipases and other lipid-active enzymes, where it has been associated with interfacial activation mechanisms (Khan et al. 2017; Bauer et al. 2020). Initially motivated by these structural and functional disparities, our study sought to dissect the structure–function relationships of mclPHA depolymerases, with the aim of elucidating how distinct architectural elements might account for their divergent catalytic behaviors. However, our rational deletions in the lid region of PhaZKT did not yield any active variants. In lipases, mutations in the lid often result in structural destabilization or complete loss of activity (Fernandez-Lopez et al. 2023; Iversen et al. 2023); however,rational engineering (whether through precise point mutations, small deletions, or domain swapping) has sometimes succeeded in enhancing catalytic performance or expanding substrate range by modifying the lid dynamics and its interaction with the active site (Karkhane et al. 2009; Yu et al. 2014; Panizza et al. 2014). The loss of activity of all mutated versions of PhaZKT designed by rational design suggests that the lid in intracellular PHA depolymerases plays an even more essential and less tolerant role than in lipases. Even single-point mutations near the lid region, such as Asn-35 (located in the core but positioned near the lid), completely abolished enzyme activity (de Eugenio et al. 2008). Together, these findings suggest the critical importance of the lid domain in PhaZKT, and, given that any alteration of the lid or its surrounding residues consistently resulted in complete loss of activity, our strategy shifted toward identifying PhaZKT variants with improved esterase activity through random mutagenesis and functional screening. Remarkably, this approach led to the identification of two active variants, one of which harbored a mutation in a region adjacent to the lid. This finding was particularly striking given the prior loss of function observed upon any rational modification of this domain. S184F is located within a hinge-like region of the lid, highlighting this domain not only as functionally sensitive but also as a potential mutational hotspot. In this position, the introduction of a sizeable aromatic side chain could lead to enhanced van der Waals interactions with the side chain of Val151 and the peptide backbone of Tyr150 and Gly186, likely influencing the interaction and relative positioning of the core and lid domains, and potentially modulating substrate accessibility. In other lipases, single amino acid substitutions in the hinge region of the lid have yielded contrasting outcomes. In some cases, hinge mutations such as D99P in *A. niger* lipase (Shu et al. 2011) or P46A in a sorghum rhizosphere microbiome esterase (Distaso et al. 2023) resulted in improved activity, presumably by adjusting lid flexibility and its dynamics. However, other substitutions have had the opposite effect; for instance, the S84G mutation in *A. niger* lipase led to a decrease in activity (Shu et al. 2011). In the case of S184F, the variant displays higher activity on soluble *p*NP esters than the wild type, with a profile more similar to that reported for extracellular depolymerases (Schirmer et al. 1993; Rhee et al. 2006; Gangoiti et al. 2012; Santos et al. 2013; Kim et al. 2003). The wild type shows maximal activity on short-chain substrates and a sharp decline with increasing chain length, resembling the intracellular depolymerase from *P. putida* LS46, which peaks with *p*NP-butyrate (C4), although activity on propionate (C3) has not been reported (Mohanan et al. 2020). Surprisingly, S184F showed a marked reduction in its ability to degrade mcl-PHAs. To investigate the structural basis underlying the effects of the S184F mutation, we performed molecular dynamics (MD) simulations, guided by previous observations in structurally related systems. Notably, a comparable hinge-like mutation (F113W) in *Rhizopus chinensis* lipase, located at a similar distance from the catalytic site (Fig. S5), was shown to promote lid flexibility and increase catalytic activity (Wang et al. 2021). In contrast, our analysis suggests that the S184F substitution may stabilize the lid region of PhaZKT rather than enhancing its dynamic opening. While this rearrangement could facilitate better access to small soluble esters, consistent with the increased esterase activity observed, it may simultaneously restrict access to bulkier polymeric substrates such as mclPHA, explaining the marked loss of depolymerase activity.

Regarding G286R, it is located within the C-terminal region of the enzyme, outside the catalytic core and distant from the lid, in a segment that remains unresolved in the available structural model. The G286R mutation may lie within an intrinsically disordered region (IDR), a flexible, unstructured segment often involved in molecular recognition or conformational regulation. IDRs, though rare in bacteria, can still mediate interactions and regulatory processes, and mutations within them may affect local dynamics with consequences for enzyme activity (Ward et al. 2004; Babu 2016; Bondos et al. 2022). Although very little is known about the role of IDRs in lipases, one study on *Bacillus* lipase showed that C-terminal deletions within an intrinsically disordered segment drastically impaired expression, secretion, and catalytic activity, underscoring the functional relevance of these regions despite the absence of a defined structure (Khurana et al. 2014). In terms of enzymatic activity, G286R behaved similarly to the wild type on short-chain *p*NP esters, except for *p*NP-octanoate (C8), where it retained higher activity, and it also exhibited superior performance on mcl-PHA substrates. This preference for C8 may help explain the improved degradation of mcl-PHA, suggesting that the same structural determinants that favor octanoate recognition may also facilitate polymer hydrolysis. The improved performance of G286R on mcl-PHA may therefore arise from improved substrate binding or altered interfacial dynamics, although the precise mechanism remains to be elucidated. In enzymes exhibiting interfacial activation, catalytic efficiency strongly depends on the physical interaction between enzyme and substrate surface and does not follow classical Michaelis–Menten behavior (Kari et al. 2017, 2018). Instead, there is often an optimal enzyme-to-substrate ratio, where both enzyme excess and insufficiency can reduce hydrolytic performance (Arnling Bååth et al. 2022). Within this framework, the broader operational range observed for G286R may reflect reduced sensitivity to enzyme loading, potentially linked to enhanced substrate accommodation or modified surface recognition. This suggests the existence of additional mutational hotspots in distal, less characterized regions of the protein.

The observed differences in the catalytic performance of both mutants on soluble and insoluble substrates also were found when analyzing the effect of different physicochemical modulators, underscoring their functional divergence. MβCD had a notably stronger inhibitory effect on G286R compared to WT and S184F, suggesting that the structural context of this mutation may confer greater sensitivity to alterations in substrate accessibility or surface interactions. Interestingly, an opposite effect of MβCD has been reported for the extracellular scl-PHA depolymerase from *Streptomyces exfoliatus*, where MβCD enhances activity (Gangoiti et al. 2012), highlighting distinct regulatory behaviors between intracellular and extracellular enzymes. In contrast, S184F retained higher activity at elevated temperatures, suggesting a modest improvement in thermal tolerance. Similar effects have been described for mutations in the lid region of the *Pseudomonas fragi* lipase (Santarossa et al. 2005). However, all variants remained equally dependent on high salt concentrations, consistent with previous observations for PhaZKT, and no substantial differences in salt sensitivity were observed among them. Finally, neither mutant nor the wild type exhibited detectable activity toward alternative polymer substrates, further supporting the idea of strong substrate specificity in this class of intracellular depolymerases.

## Conclusions

In this work, we aimed to investigate the influence of the lid region on the differences between intracellular and extracellular PHA depolymerases, and to explore strategies to improve enzymatic activity in PhaZKT. Our results show that targeted modifications within the lid region were detrimental, leading to a loss of depolymerase activity. However, using random mutagenesis, two mutants with increased activity were obtained. In S184F, the mutation was located near the lid and resulted in higher esterase activity but almost complete loss of depolymerase activity, underscoring the essential role of lid integrity. In contrast, G286R, with a mutation in a region distant from the lid, showed improved activity toward mclPHA while also increasing esterase activity. Molecular dynamics simulations indicated that the lid in S184F is slightly more rigid compared to the wild type, potentially explaining its altered substrate specificity. Overall, these findings suggest that the lid is critical and highly sensitive for PHA hydrolysis, while distal regions may provide good targets for engineering improved enzyme activity without compromising depolymerase function.

Supplementary information

## Supplementary Information

Below is the link to the electronic supplementary material.

ESM1 (PPTX.9.60 MB)

ESM2 (DOCX.16.6 KB)

ESM3 (DOCX.17.4 KB)

ESM4 (PPTX.11.9 MB)

## Data Availability

The microbial strains engineered in this study are stored in our institutional strain collection and may be provided upon justified request and under appropriate material transfer agreements.
