# Supplementary material for: Revealing the essential role of the lid in mclPHA intracellular depolymerase from Pseudomonas putida KT2440
Source: Appl Microbiol Biotechnol. 2025 Oct 7;109:215. doi: 10.1007/s00253-025-13605-z (PMC12504323; doi:10.1007/s00253-025-13605-z)
Supplement: Supplementary file 1 [file 253_2025_13605_MOESM2_ESM.docx]

Table S1. Influence of salt and additives on the relative activity (%) of PhaZKT and its variants

PhaZKT	G286R	S184F

NaCl

300 mM	100.0 ± 3.5	100.0 ± 0.8	100.0 ± 1.9

60 mM	68.7 ± 9.1	57.7 ± 15.2	62.5 ± 7.0

Sarkosyl 0.02%	31.45 ± 1.0	33.74 ± 1.6	32.1 ± 1.6

Methyl-β-cyclodextrin 5%	65.4 ± 3.8	40.8 ± 1.2	65.3 ± 2.6

Data represent mean ± Standard Deviation of triplicate experiments.
