# Supplementary material for: Revealing the essential role of the lid in mclPHA intracellular depolymerase from Pseudomonas putida KT2440
Source: Appl Microbiol Biotechnol. 2025 Oct 7;109:215. doi: 10.1007/s00253-025-13605-z (PMC12504323; doi:10.1007/s00253-025-13605-z)
Supplement: Supplementary file 2 [file 253_2025_13605_MOESM3_ESM.docx]

Table S2: Comparison of residue-specific RMSF values between PhaZKT and S184F

aa	PhaZKT	S184F	ΔRSMF

1	455	454	1

2	304	336	-32

3	310	319	-9

4	356	354	2

5	254	261	-7

6	366	372	-6

7	262	277	-15

8	347	351	-4

9	339	342	-3

10	357	351	6

11	285	287	-2

12	362	359	3

13	291	269	22

14	328	335	-7

15	264	257	7

16	225	224	1

17	350	343	7

18	235	244	-9

19	309	301	8

20	309	312	-3

21	369	372	-3

22	240	233	7

23	344	326	18

24	293	283	10

25	363	337	26

26	286	286	0

27	293	290	3

28	385	388	-3

29	317	320	-3

30	340	325	15

31	344	345	-1

32	350	340	10

33	336	339	-3

34	223	226	-3

35	244	260	-16

36	223	236	-13

37	337	340	-3

38	232	249	-17

39	306	328	-22

40	220	219	1

41	321	326	-5

42	244	240	4

43	339	352	-13

44	350	351	-1

45	268	259	9

46	250	247	3

47	243	256	-13

48	330	318	12

49	260	271	-11

50	318	330	-12

51	349	348	1

52	286	275	11

53	267	292	-25

54	323	297	26

55	346	344	2

56	246	243	3

57	345	332	13

58	404	354	50

59	310	315	-5

60	234	244	-10

61	206	218	-12

62	342	352	-10

63	233	219	14

64	227	239	-12

65	349	344	5

66	241	243	-2

67	259	252	7

68	238	230	8

69	223	233	-10

70	321	324	-3

71	277	249	28

72	354	376	-22

73	322	349	-27

74	274	326	-52

75	230	225	5

76	271	266	5

77	218	220	-2

78	255	254	1

79	217	219	-2

80	339	346	-7

81	286	295	-9

82	337	345	-8

83	355	366	-11

84	293	281	12

85	294	294	0

86	294	252	42

87	312	321	-9

88	343	346	-3

89	274	277	-3

90	293	285	8

91	374	362	12

92	310	401	-91

93	265	265	0

94	273	297	-24

95	318	303	15

96	354	360	-6

97	240	274	-34

98	350	353	-3

99	334	329	5

100	220	211	9

101	354	359	-5

102	225	230	-5

103	216	213	3

104	187	187	0

105	197	195	2

106	293	296	-3

107	308	331	-23

108	281	275	6

109	226	218	8

110	231	231	0

111	248	251	-3

112	299	309	-10

113	255	255	0

114	255	247	8

115	294	266	28

116	253	241	12

117	259	287	-28

118	235	246	-11

119	370	244	126

120	335	347	-12

121	320	337	-17

122	347	345	2

123	326	332	-6

124	347	343	4

125	293	292	1

126	295	292	3

127	286	284	2

128	318	323	-5

129	338	343	-5

130	258	262	-4

131	350	367	-17

132	372	378	-6

133	386	370	16

134	364	356	8

135	284	259	25

136	236	240	-4

137	347	440	-93

138	271	285	-14

139	355	361	-6

140	337	342	-5

141	346	365	-19

142	279	274	5

143	313	337	-24

144	334	357	-23

145	360	357	3

146	277	276	1

147	263	269	-6

148	320	329	-9

149	348	331	17

150	222	226	-4

151	368	350	18

152	312	294	18

153	283	253	30

154	288	276	12

155	253	253	0

156	340	340	0

157	350	356	-6

158	350	327	23

159	338	330	8

160	306	304	2

161	276	322	-46

162	322	347	-25

163	341	344	-3

164	232	251	-19

165	227	229	-2

166	206	229	-23

167	214	216	-2

168	239	248	-9

169	286	366	-80

170	267	311	-44

171	289	285	4

172	269	272	-3

173	287	283	4

174	360	353	7

175	302	304	-2

176	354	364	-10

177	304	327	-23

178	308	282	26

179	300	308	-8

180	339	342	-3

181	432	315	117

182	362	364	-2

183	334	335	-1

184	282	263	19

185	261	321	-60

186	262	268	-6

187	333	333	0

188	353	349	4

189	217	217	0

190	232	223	9

191	265	275	-10

192	218	227	-9

193	213	228	-15

194	345	344	1

195	263	255	8

196	312	311	1

197	254	263	-9

198	390	381	9

199	269	266	3

200	222	221	1

201	328	296	32

202	207	210	-3

203	354	356	-2

204	263	255	8

205	210	214	-4

206	321	336	-15

207	267	270	-3

208	343	343	0

209	357	354	3

210	321	313	8

211	283	266	17

212	252	254	-2

213	273	294	-21

214	342	336	6

215	337	332	5

216	352	330	22

217	319	293	26

218	237	238	-1

219	321	332	-11

220	246	312	-66

221	217	225	-8

222	233	315	-82

223	351	349	2

224	337	336	1

225	241	227	14

226	348	352	-4

227	352	346	6

228	220	223	-3

229	364	284	80

230	274	281	-7

231	368	369	-1

232	342	341	1

233	312	306	6

234	268	272	-4

235	334	316	18

236	327	331	-4

237	273	269	4

238	307	289	18

239	325	332	-7

240	327	361	-34

241	356	356	0

242	283	275	8

243	358	361	-3

244	357	381	-24

245	262	322	-60

246	216	226	-10

247	226	222	4

248	221	212	9

249	372	369	3

250	218	220	-2

251	349	347	2

252	369	366	3

253	289	319	-30

254	314	318	-4

255	301	326	-25

256	327	334	-7

257	338	335	3

258	356	367	-11

259	293	303	-10

260	280	325	-45

261	360	353	7

262	350	354	-4

263	319	322	-3

264	383	384	-1

265	276	268	8
