# Supplementary material for: Revealing the essential role of the lid in mclPHA intracellular depolymerase from Pseudomonas putida KT2440
Source: Appl Microbiol Biotechnol. 2025 Oct 7;109(1):215. doi: 10.1007/s00253-025-13605-z (PMC12504323; doi:10.1007/s00253-025-13605-z)
Supplement: Supplementary file 1 — (PPTX.9.60 MB) [file 253_2025_13605_MOESM1_ESM.pptx]

## Slide 1
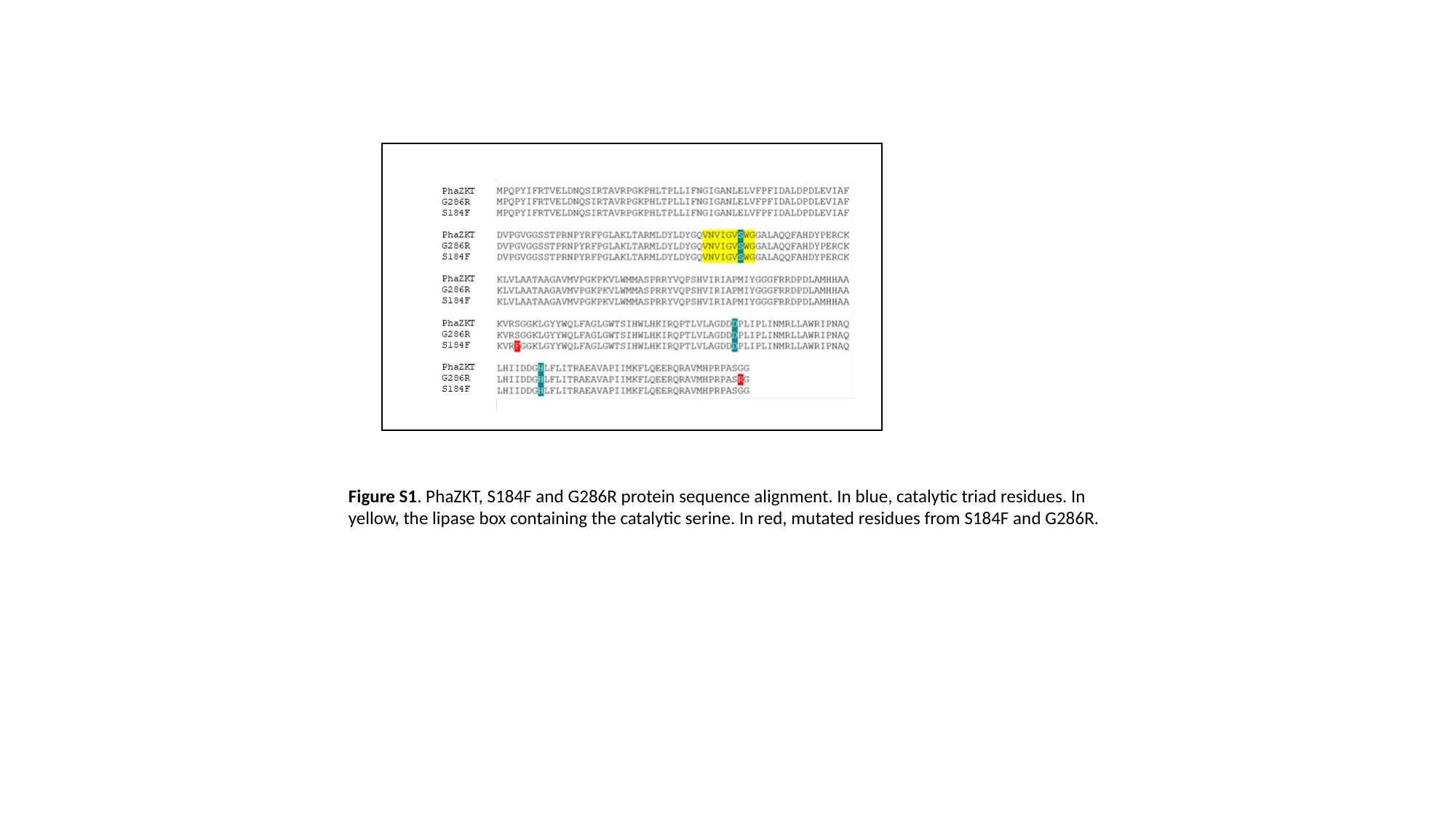

Figure S1. PhaZKT, S184F and G286R protein sequence alignment. In blue, catalytic triad residues. In yellow, the lipase box containing the catalytic serine. In red, mutated residues from S184F and G286R.

## Slide 2
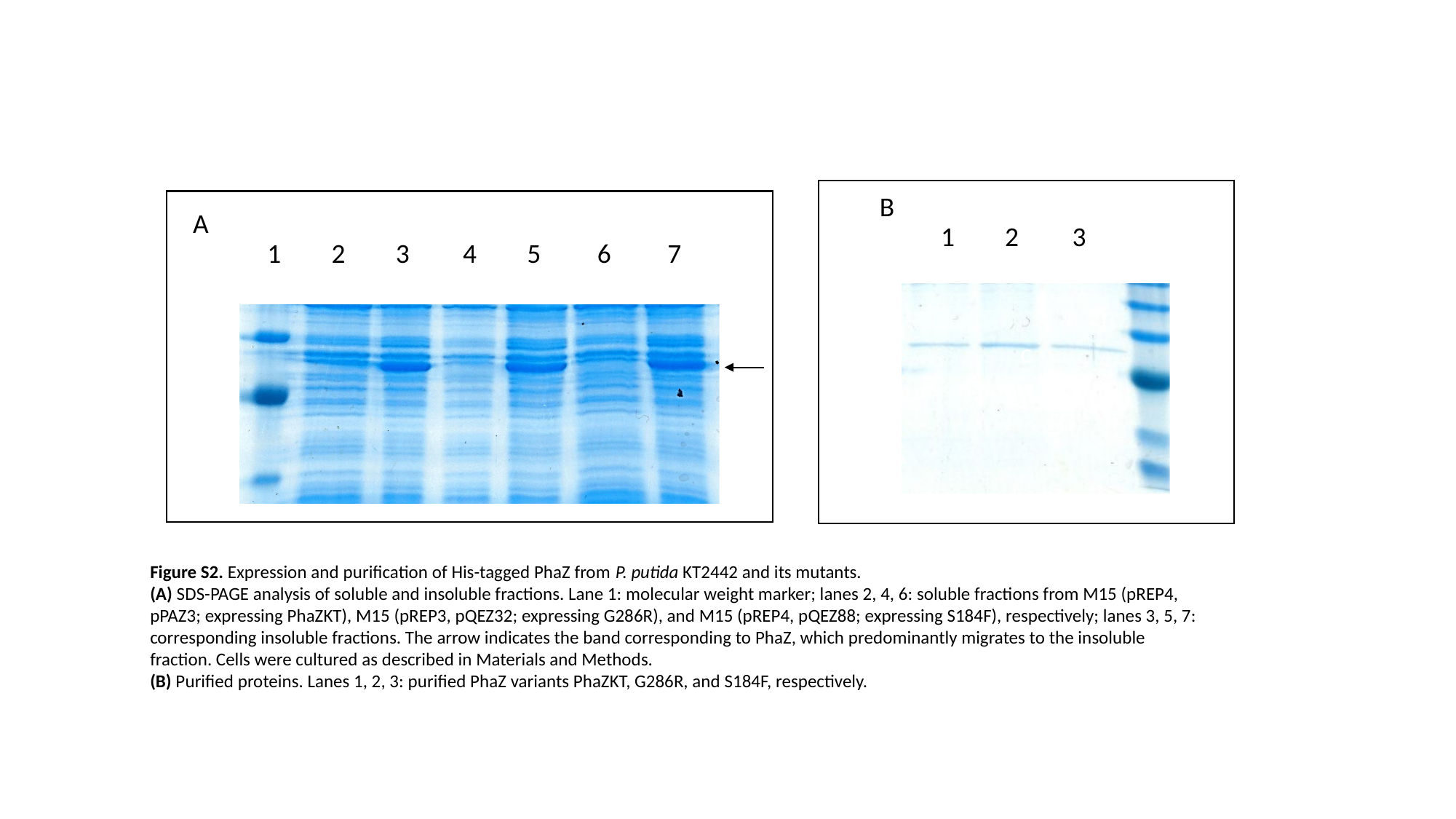

c
B
A
1
2
3
1
2
3
4
5
6
7
Figure S2. Expression and purification of His-tagged PhaZ from P. putida KT2442 and its mutants.
(A) SDS-PAGE analysis of soluble and insoluble fractions. Lane 1: molecular weight marker; lanes 2, 4, 6: soluble fractions from M15 (pREP4, pPAZ3; expressing PhaZKT), M15 (pREP3, pQEZ32; expressing G286R), and M15 (pREP4, pQEZ88; expressing S184F), respectively; lanes 3, 5, 7: corresponding insoluble fractions. The arrow indicates the band corresponding to PhaZ, which predominantly migrates to the insoluble fraction. Cells were cultured as described in Materials and Methods.
(B) Purified proteins. Lanes 1, 2, 3: purified PhaZ variants PhaZKT, G286R, and S184F, respectively.

## Slide 3
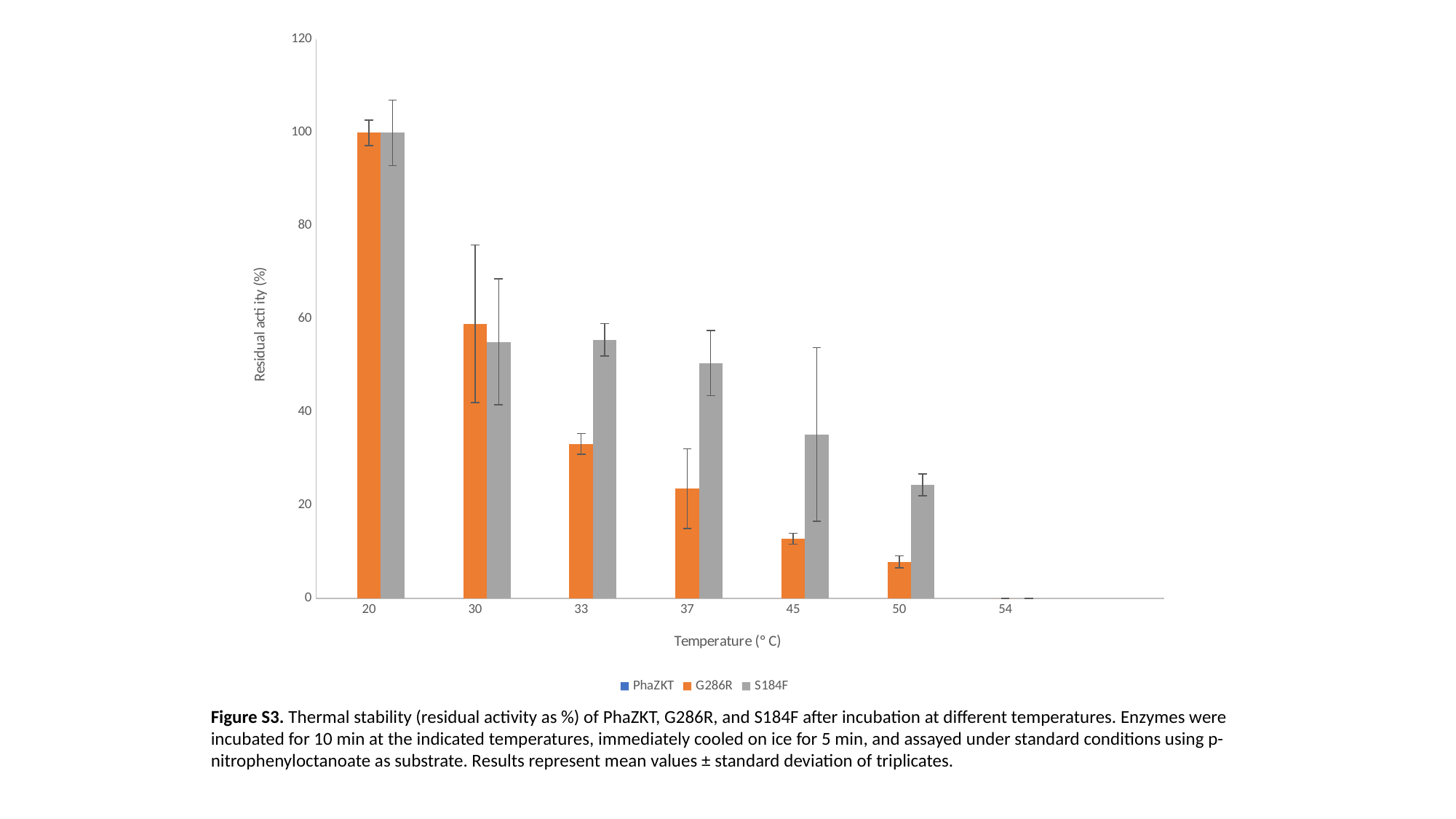

### Chart
| Category | | | |
|---|---|---|---|
| 20 | 100.0 | 100.0 | 100.0 |
| 30 | 60.225965575887884 | 59.007465200430275 | 55.101875103974386 |
| 33 | 28.47363852564891 | 33.190259380399205 | 55.56629407524179 |
| 37 | 22.240884102665646 | 23.547437165179748 | 50.541062955011725 |
| 45 | 9.997580695511038 | 12.829283329433725 | 35.21575771376524 |
| 50 | 8.226025472935003 | 7.853033696180992 | 24.378016761029407 |
| 54 | 0.0 | 0.0 | 0.0 |Figure S3. Thermal stability (residual activity as %) of PhaZKT, G286R, and S184F after incubation at different temperatures. Enzymes were incubated for 10 min at the indicated temperatures, immediately cooled on ice for 5 min, and assayed under standard conditions using p-nitrophenyloctanoate as substrate. Results represent mean values ± standard deviation of triplicates.

## Slide 4
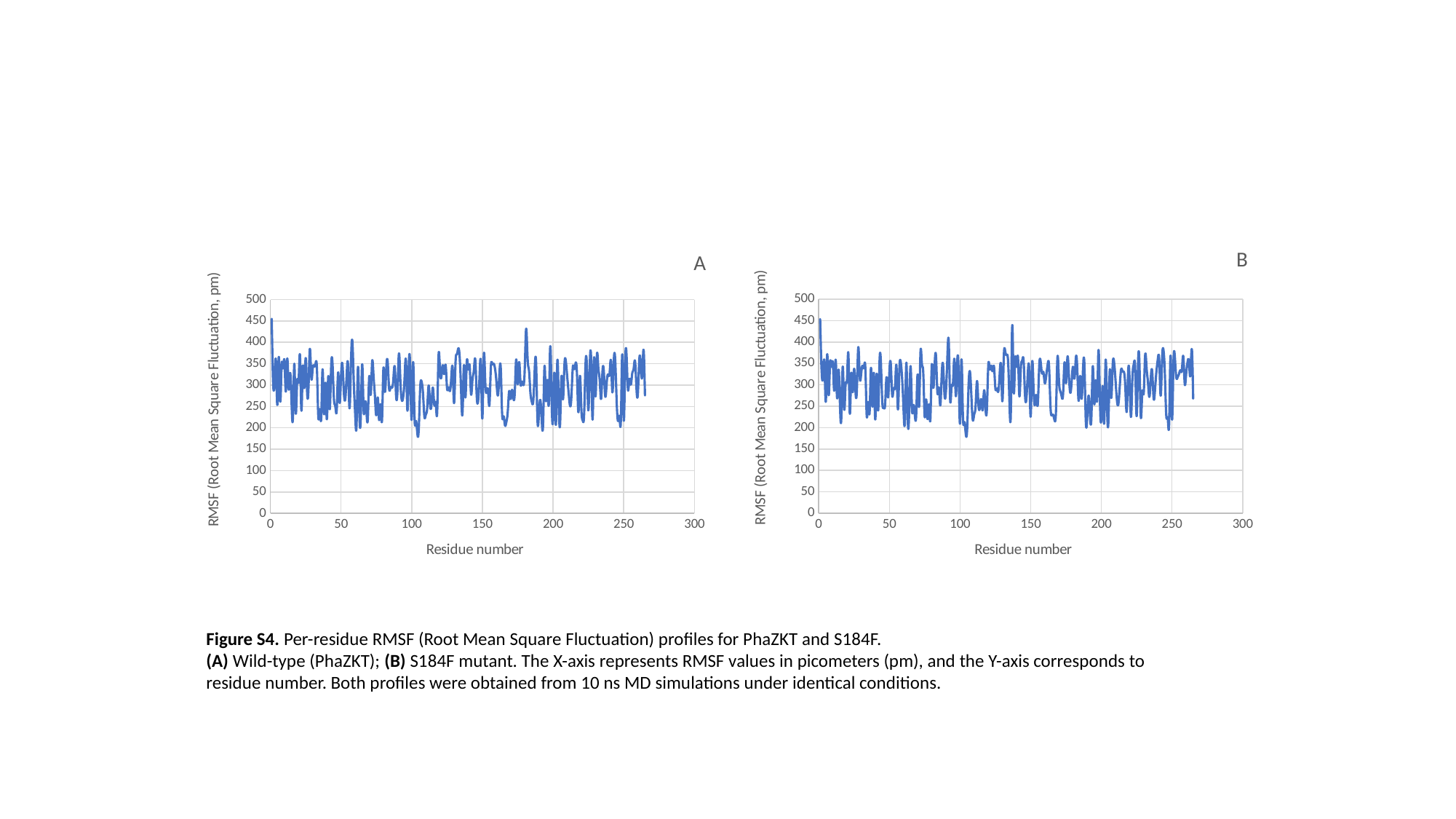

### Chart: B
| Category | |
|---|---|
### Chart: A
| Category | Column1.2.2 |
|---|---|Figure S4. Per-residue RMSF (Root Mean Square Fluctuation) profiles for PhaZKT and S184F.
(A) Wild-type (PhaZKT); (B) S184F mutant. The X-axis represents RMSF values in picometers (pm), and the Y-axis corresponds to residue number. Both profiles were obtained from 10 ns MD simulations under identical conditions.

## Slide 5
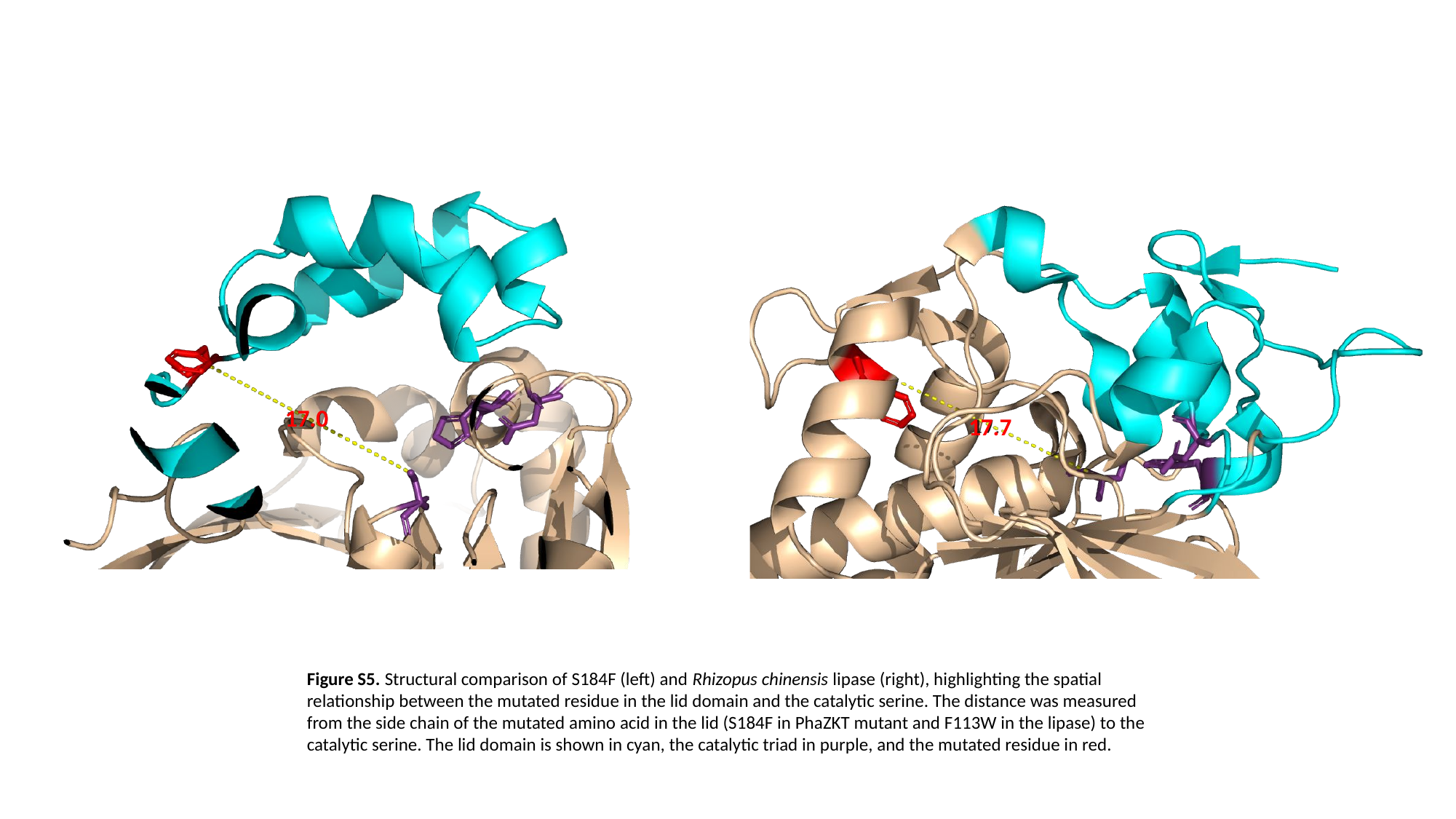

17.0
17.7
Figure S5. Structural comparison of S184F (left) and Rhizopus chinensis lipase (right), highlighting the spatial relationship between the mutated residue in the lid domain and the catalytic serine. The distance was measured from the side chain of the mutated amino acid in the lid (S184F in PhaZKT mutant and F113W in the lipase) to the catalytic serine. The lid domain is shown in cyan, the catalytic triad in purple, and the mutated residue in red.
