# Supplementary material for: Revealing the essential role of the lid in mclPHA intracellular depolymerase from Pseudomonas putida KT2440
Source: Appl Microbiol Biotechnol. 2025 Oct 7;109(1):215. doi: 10.1007/s00253-025-13605-z (PMC12504323; doi:10.1007/s00253-025-13605-z)
Supplement: Supplementary file 2 — (DOCX.16.6 KB) [file 253_2025_13605_MOESM2_ESM.docx]

Table S1. Influence of salt and additives on the relative activity (%) of PhaZKT and its variants

PhaZKT G286R S184F

NaCl

300 mM 100.0 ± 3.5 100.0 ± 0.8 100.0 ± 1.9

60 mM 68.7 ± 9.1 57.7 ± 15.2 62.5 ± 7.0

Sarkosyl 0.02% 31.45 ± 1.0 33.74 ± 1.6 32.1 ± 1.6

Methyl-β-cyclodextrin 5% 65.4 ± 3.8 40.8 ± 1.2 65.3 ± 2.6

Data represent mean ± Standard Deviation of triplicate experiments.
