# Supplementary material for: Revealing the essential role of the lid in mclPHA intracellular depolymerase from Pseudomonas putida KT2440
Source: Appl Microbiol Biotechnol. 2025 Oct 7;109(1):215. doi: 10.1007/s00253-025-13605-z (PMC12504323; doi:10.1007/s00253-025-13605-z)
Supplement: Supplementary file 3 — (DOCX.17.4 KB) [file 253_2025_13605_MOESM3_ESM.docx]

Table S2: Comparison of residue-specific RMSF values between PhaZKT and S184F

aa PhaZKT S184F ΔRSMF

1 455 454 1

2 304 336 -32

3 310 319 -9

4 356 354 2

5 254 261 -7

6 366 372 -6

7 262 277 -15

8 347 351 -4

9 339 342 -3

10 357 351 6

11 285 287 -2

12 362 359 3

13 291 269 22

14 328 335 -7

15 264 257 7

16 225 224 1

17 350 343 7

18 235 244 -9

19 309 301 8

20 309 312 -3

21 369 372 -3

22 240 233 7

23 344 326 18

24 293 283 10

25 363 337 26

26 286 286 0

27 293 290 3

28 385 388 -3

29 317 320 -3

30 340 325 15

31 344 345 -1

32 350 340 10

33 336 339 -3

34 223 226 -3

35 244 260 -16

36 223 236 -13

37 337 340 -3

38 232 249 -17

39 306 328 -22

40 220 219 1

41 321 326 -5

42 244 240 4

43 339 352 -13

44 350 351 -1

45 268 259 9

46 250 247 3

47 243 256 -13

48 330 318 12

49 260 271 -11

50 318 330 -12

51 349 348 1

52 286 275 11

53 267 292 -25

54 323 297 26

55 346 344 2

56 246 243 3

57 345 332 13

58 404 354 50

59 310 315 -5

60 234 244 -10

61 206 218 -12

62 342 352 -10

63 233 219 14

64 227 239 -12

65 349 344 5

66 241 243 -2

67 259 252 7

68 238 230 8

69 223 233 -10

70 321 324 -3

71 277 249 28

72 354 376 -22

73 322 349 -27

74 274 326 -52

75 230 225 5

76 271 266 5

77 218 220 -2

78 255 254 1

79 217 219 -2

80 339 346 -7

81 286 295 -9

82 337 345 -8

83 355 366 -11

84 293 281 12

85 294 294 0

86 294 252 42

87 312 321 -9

88 343 346 -3

89 274 277 -3

90 293 285 8

91 374 362 12

92 310 401 -91

93 265 265 0

94 273 297 -24

95 318 303 15

96 354 360 -6

97 240 274 -34

98 350 353 -3

99 334 329 5

100 220 211 9

101 354 359 -5

102 225 230 -5

103 216 213 3

104 187 187 0

105 197 195 2

106 293 296 -3

107 308 331 -23

108 281 275 6

109 226 218 8

110 231 231 0

111 248 251 -3

112 299 309 -10

113 255 255 0

114 255 247 8

115 294 266 28

116 253 241 12

117 259 287 -28

118 235 246 -11

119 370 244 126

120 335 347 -12

121 320 337 -17

122 347 345 2

123 326 332 -6

124 347 343 4

125 293 292 1

126 295 292 3

127 286 284 2

128 318 323 -5

129 338 343 -5

130 258 262 -4

131 350 367 -17

132 372 378 -6

133 386 370 16

134 364 356 8

135 284 259 25

136 236 240 -4

137 347 440 -93

138 271 285 -14

139 355 361 -6

140 337 342 -5

141 346 365 -19

142 279 274 5

143 313 337 -24

144 334 357 -23

145 360 357 3

146 277 276 1

147 263 269 -6

148 320 329 -9

149 348 331 17

150 222 226 -4

151 368 350 18

152 312 294 18

153 283 253 30

154 288 276 12

155 253 253 0

156 340 340 0

157 350 356 -6

158 350 327 23

159 338 330 8

160 306 304 2

161 276 322 -46

162 322 347 -25

163 341 344 -3

164 232 251 -19

165 227 229 -2

166 206 229 -23

167 214 216 -2

168 239 248 -9

169 286 366 -80

170 267 311 -44

171 289 285 4

172 269 272 -3

173 287 283 4

174 360 353 7

175 302 304 -2

176 354 364 -10

177 304 327 -23

178 308 282 26

179 300 308 -8

180 339 342 -3

181 432 315 117

182 362 364 -2

183 334 335 -1

184 282 263 19

185 261 321 -60

186 262 268 -6

187 333 333 0

188 353 349 4

189 217 217 0

190 232 223 9

191 265 275 -10

192 218 227 -9

193 213 228 -15

194 345 344 1

195 263 255 8

196 312 311 1

197 254 263 -9

198 390 381 9

199 269 266 3

200 222 221 1

201 328 296 32

202 207 210 -3

203 354 356 -2

204 263 255 8

205 210 214 -4

206 321 336 -15

207 267 270 -3

208 343 343 0

209 357 354 3

210 321 313 8

211 283 266 17

212 252 254 -2

213 273 294 -21

214 342 336 6

215 337 332 5

216 352 330 22

217 319 293 26

218 237 238 -1

219 321 332 -11

220 246 312 -66

221 217 225 -8

222 233 315 -82

223 351 349 2

224 337 336 1

225 241 227 14

226 348 352 -4

227 352 346 6

228 220 223 -3

229 364 284 80

230 274 281 -7

231 368 369 -1

232 342 341 1

233 312 306 6

234 268 272 -4

235 334 316 18

236 327 331 -4

237 273 269 4

238 307 289 18

239 325 332 -7

240 327 361 -34

241 356 356 0

242 283 275 8

243 358 361 -3

244 357 381 -24

245 262 322 -60

246 216 226 -10

247 226 222 4

248 221 212 9

249 372 369 3

250 218 220 -2

251 349 347 2

252 369 366 3

253 289 319 -30

254 314 318 -4

255 301 326 -25

256 327 334 -7

257 338 335 3

258 356 367 -11

259 293 303 -10

260 280 325 -45

261 360 353 7

262 350 354 -4

263 319 322 -3

264 383 384 -1

265 276 268 8
