# Supplementary material for: Revealing the essential role of the lid in mclPHA intracellular depolymerase from Pseudomonas putida KT2440
Source: Appl Microbiol Biotechnol. 2025 Oct 7;109(1):215. doi: 10.1007/s00253-025-13605-z (PMC12504323; doi:10.1007/s00253-025-13605-z)
Supplement: Supplementary file 4 — (PPTX.11.9 MB) [file 253_2025_13605_MOESM4_ESM.pptx]

## Slide 1
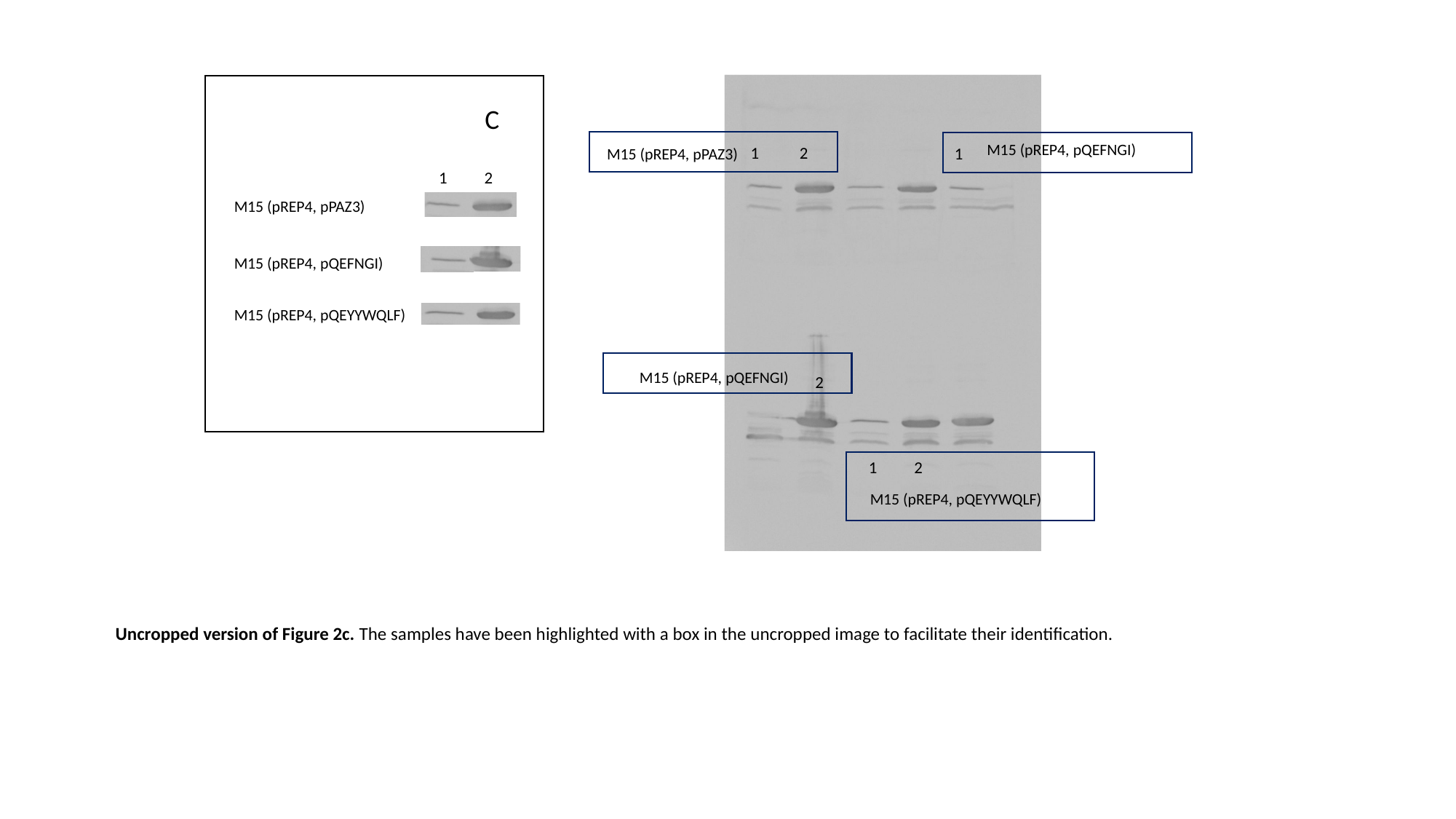

C
M15 (pREP4, pQEFNGI)
1
2
1
M15 (pREP4, pPAZ3)
1
2
M15 (pREP4, pPAZ3)
M15 (pREP4, pQEFNGI)
M15 (pREP4, pQEYYWQLF)
M15 (pREP4, pQEFNGI)
2
1
2
M15 (pREP4, pQEYYWQLF)
Uncropped version of Figure 2c. The samples have been highlighted with a box in the uncropped image to facilitate their identification.

## Slide 2
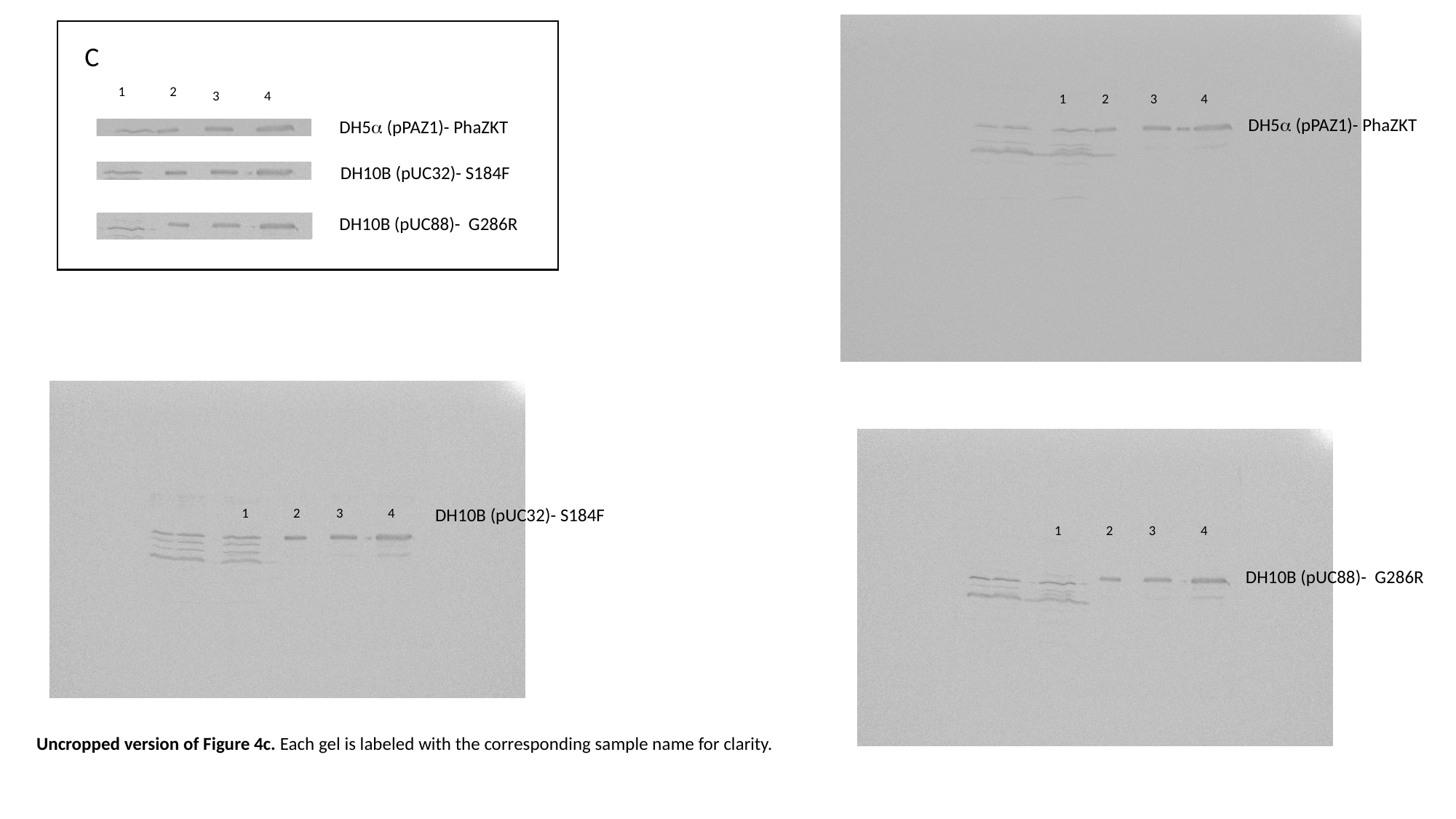

c
C
1
2
3
4
1
2
3
4
DH5a (pPAZ1)- PhaZKT
DH5a (pPAZ1)- PhaZKT
DH10B (pUC32)- S184F
DH10B (pUC88)- G286R
DH10B (pUC32)- S184F
1
2
3
4
1
2
3
4
DH10B (pUC88)- G286R
Uncropped version of Figure 4c. Each gel is labeled with the corresponding sample name for clarity.

## Slide 3
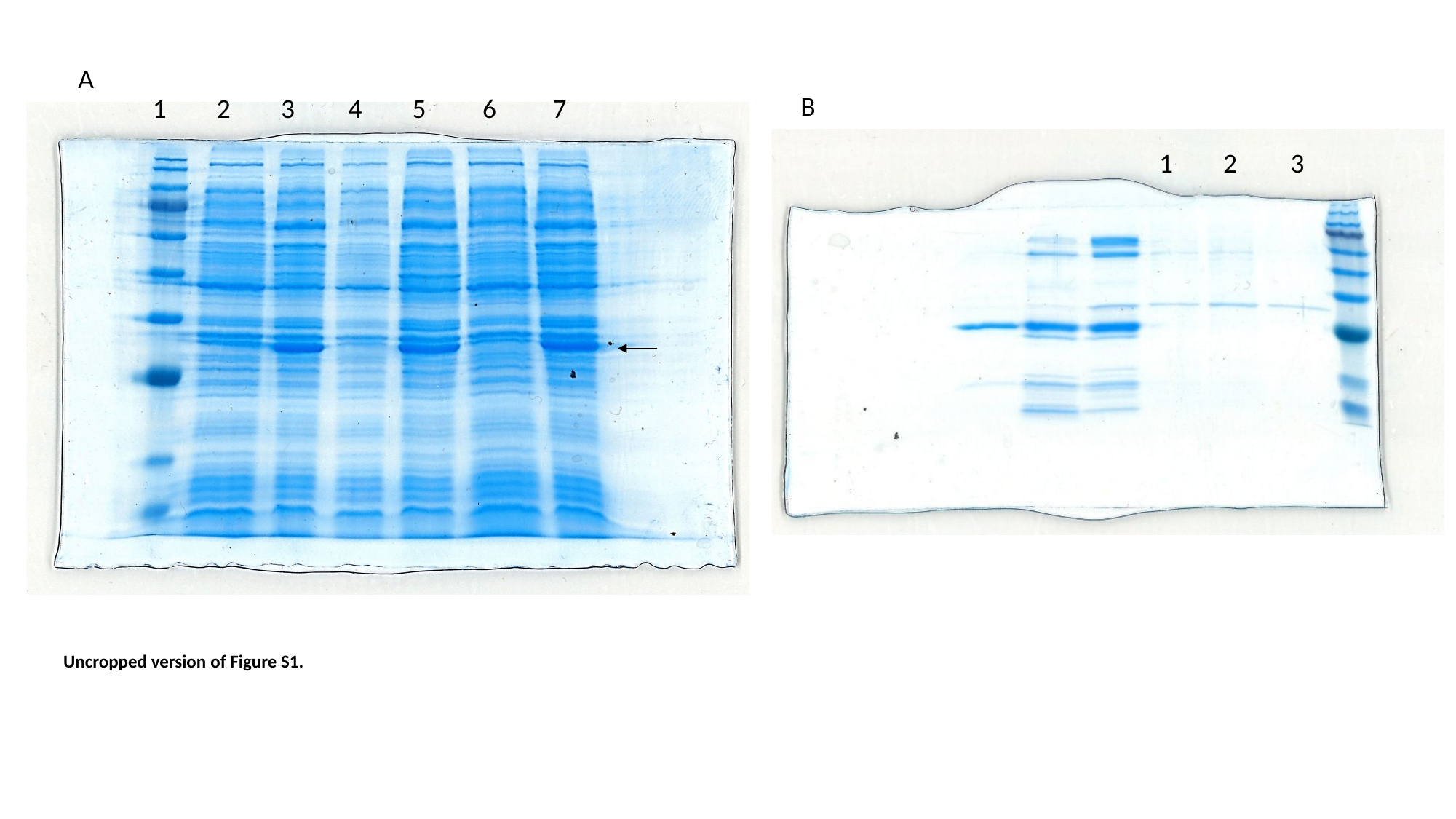

A
B
1
2
3
4
5
6
7
1
2
3
Uncropped version of Figure S1.
